# Vaccination status of individuals with diabetes mellitus treated in Primary Healthcare: a cross-sectional study*

**DOI:** 10.1590/1518-8345.7065.4452

**Published:** 2025-02-03

**Authors:** Francisco João de Carvalho, Luisa Helena de Oliveira Lima, Mônica Antar Gamba, Rosilane Lima de Brito, Lucilane Maria Sales da Silva, Ana Roberta Vilarouca da Silva

**Affiliations:** 1 Universidade Estadual do Ceará, Fortaleza, CE, Brazil.; 2 Scholarship holder at the Fundação Cearense de Apoio ao Desenvolvimento Científico e Tecnológico (FUNCAP), Brazil.; 3 Universidade Federal do Piauí, Teresina, PI, Brazil.; 4 Universidade Federal de São Paulo, São Paulo, SP, Brazil.

**Keywords:** Vaccination Coverage, Diabetes Mellitus, Type 2, Diabetes Mellitus, Type 1, Primary Health Care, Nursing, Vaccines

## Abstract

to analyze the vaccination status of individuals with type 1 and type 2 diabetes in Primary Healthcare.

cross-sectional, analytical study conducted in 25 Family Health teams with 274 individuals with diabetes. Sociodemographic and clinical variables were evaluated, as well as the full vaccination schedule for each vaccine recommended by the National Immunization Program, through bivariate analysis and logistic regression.

among individuals with diabetes mellitus, the following incomplete vaccination rates were found: 69.1% for hepatitis B; 64.6% for diphtheria and tetanus; 74.3% for yellow fever; 87.9% for pneumococcus; 87.9% for varicella; 24.5% for influenza; and 0.7% for COVID-19. The reported reasons for low vaccination rates included not knowing the importance of vaccination and not being informed by healthcare providers. A statistically significant association was found between sociodemographic and clinical profile regarding the full vaccination schedule between the influenza vaccine and age and income; COVID-19 and age, type of diabetes and duration of diabetes.

individuals with type 1 and type 2 diabetes treated in Primary Healthcare showed low vaccination rates, which is concerning due to increased vulnerability to vaccine-preventable infections and mortality within this group.

## Highlights

**(1)** Individuals with diabetes have low vaccination rates.

**(2)** It is essential to provide vaccination opportunities in health services for individuals with diabetes.

**(3)** Education for vaccine-prescribing professionals is necessary.

**(4)** Young people are less likely to get vaccinated than older adults.

**(5)** It is essential to understand the predisposing factors related to vaccination status.

## Introduction

It is estimated that 537 million adults aged 20 to 79 worldwide have diabetes mellitus (DM). By 2030, the estimate is 643 million, and by 2045, 783 million. Brazil features among the top 10 countries in number of adults (ages 20 to 79) with DM in 2021, ranking 6th^(1)^.

This persistent hyperglycemic condition significantly interferes with the effectiveness of the immune system in these individuals, as it compromises the defense mechanisms, including suppression of cytokine production, defects in phagocytosis, dysfunction of immune cells and an inability to fight microorganisms^(2-3)^. This makes them more susceptible to certain infectious diseases^(3)^ that could be prevented through vaccination, hindering the reduction of morbidity and hospitalization^(4)^.

Immunization of individuals with DM is a fundamental strategy for health protection and promotion of quality of life. It should be noted that they require specialized attention, as they have: 2.5 times higher risk for developing severe acute respiratory syndrome (SARS) from influenza; 1.4 times higher risk for community-acquired pneumonia; 4.6 times higher risk for invasive pneumococcal disease; twice the risk for liver complications when also having chronic hepatitis B; and three times higher risk for herpes zoster.

In accordance with the Brazilian Society of Diabetes (SBD) and the Brazilian Society of Immunization (SBIm), and considering the recommendations from the Special Immunobiology Referral Centers (CRIE), the following vaccines are highly recommended for individuals with diabetes: influenza, 13-valent conjugate pneumococcal (PCV13), 23-valent polysaccharide pneumococcal (PPSV23), hepatitis B, *Haemophilus influenzae* type b (Hib), varicella and herpes zoster, noting that most are available in the National Immunization Program (PNI).

Among the vaccines available in Primary Healthcare (APS) and the CRIEs are vaccines against influenza, hepatitis B, yellow fever, adult diphtheria and tetanus (dT), measles-mumps-rubella (MMR) and COVID-19. Although the PPSV23, PCV13 and varicella pneumococcal vaccines are recommended, they are not available in APS for individuals with DM, only in the CRIEs with a medical prescription. In turn, the herpes zoster vaccine is available only in the private sector.

Vaccination is an essential pillar of APS and an unquestionable human right. The APS network centers verify vaccination records and status and provide guidance and administration to initiate or update the vaccination schedule according to specific calendars^(7)^. Furthermore, the detailed recording of vaccines received by individuals with DM, along with the assessment of their needs, are essential elements of a holistic clinical evaluation conducted at the first consultation and during annual follow-up^(7)^.

Social determinants influence the decision to get vaccinated. Among the socioeconomic and clinical factors associated with vaccination status, lower educational level and income negatively impact vaccination rates, while older age is associated with higher vaccination rates^(8)^. In addition, factors related to logistics and healthcare providers professionals may pose barriers to vaccination^(9)^.

However, despite the recommendations, Brazilian^(10-11)^ and foreign^(12-14)^ studies assessing vaccination in individuals with DM did not include all the vaccines recommended for this group. In addition to filling this gap, this survey also addresses the perceptions of individuals with DM regarding vaccination as well as the difficulties they face in getting vaccinated. In light of this, planning and implementing strategies to reach the population with DM are essential and urgent. Therefore, the goal was to analyze the vaccination status of individuals with type 1 and type 2 diabetes treated in Primary Healthcare.

## Method

### Type of study

Cross-sectional, analytical study based on the guidelines of Strengthening the Reporting of Observational Studies in Epidemiology (STROBE)^(15)^.

### Study site

The survey was conducted in a municipality in Piauí, Brazil, located in the mid-southern region of the state, with an estimated population of 83,000. APS covers 100% of the municipality and comprises 36 Family Health teams (FHTs), 25 of them in the urban area and 11 in rural areas. These teams make up the Family Health units (FHUs), whose facilities include at least one FHT.

### Period

The data were collected between January and July 2022.

### Population and sample

The population consisted of patients registered with the 25 FHT of the urban area of the studied municipality. The information system used for registering individuals with DM is the e-SUS APS. Sample calculation was performed for a finite population, stratified by proportion, totaling 2,564 individuals with type 1 DM (DM1) and type 2 DM (DM2), with a sampling error of 5% and a confidence level of 95%. For calculating prevalence, a value of 0.5 was used to achieve the maximum sample size, considering a 95% confidence level and a 5% significance level. One advantage of the proportional stratified sampling was the proportional distribution of registered individuals with DM according to the population size of each FHT. Thus, the final sample consisted of 274 individuals with DM.

### Selection criteria

The sample included individuals aged 18 or older, diagnosed with either DM1 or DM2 and registered with the FHUs for at least one year. Individuals who exhibited any cognitive deficit that prevented them from directly answering the questions in the data collection instrument, assessed through the administration of the Mini-Mental State Examination, were excluded.

### Data collection

This stage was conducted through the administration of a data collection form during nursing consultations with individuals with DM who agreed to participate in the research by signing the Informed Consent Form (ICF). Home visits were conducted for individuals who did not frequent the FHUs, accompanied by community health agents (ACS). The process involved the following steps: 1. Meetings were held with the ACS and nurses in which participants were drawn based on the available information about individuals with DM in each FHT. 2. The phones numbers of the selected individuals were requested in order to compose the sample. 3. A home visit was scheduled by phone, in which the ICF and household entry agreement were explained. 4. Those who agreed to participate were asked to provide an appropriate environment in the home for the administration of the data collection instrument. 5. The household entry agreement was used to access the residence. Due to the COVID-19 pandemic, protective measures were implemented to ensure the safety of both researchers and interviewees.

### Collection instrument and study variables

A data collection instrument titled “Vaccination Status of Individuals with Diabetes in Primary Healthcare” was designed by the researcher, based on the recommendations from the SBD and the SBIm, as well as a literature review on the subject. It contains 30 items, with multiple-choice and open-ended questions, divided into three dimensions. Sociodemographic and clinical characteristics (16 items) include variables such as gender, level of education, income, skin color, religion, occupation, type of DM, duration of diabetes in years, medication and non-medication treatments, comorbidities, adherence to medication, smoking and alcohol consumption habits, and regular physical exercise (more than three times a week).

The vaccination status dimension (three items) assesses whether the individual has a vaccination card and produced it, the immunization status for hepatitis B, influenza, dT, MMR, yellow fever, COVID-19, varicella and conjugate pneumococcal vaccines, and where the vaccines were administered (FHUs, secondary care, home or CRIE).

The last dimension concerns the perception of individuals with DM regarding vaccination (11 items), which included: knowledge about the PNI; whether they consider the media campaigns conducted by the PNI sufficient to motivate vaccination; reasons for having an incomplete vaccination status (fear of experiencing adverse reactions, lack of awareness about the importance of vaccination, previous experiences of reactions to certain vaccines, not deeming it necessary, lack of information from healthcare providers, and other reasons - please specify). Participants could provide more than one answer. Additionally, the survey asked about guidance from healthcare providers regarding the importance of keeping up to date with the vaccination schedule; adherence to vaccination guidance provided by healthcare providers; visits to the FHUs to complete the vaccination schedule s requiring two or more doses; adverse effects following vaccination; health problems that prevented them from getting vaccinated; medical or nursing advice against receiving further vaccines after such health problems; difficulties in getting vaccinated (FHUs being too far away, lack of time, inconvenient operating hours of the vaccination site, being told to return another day, not considering it necessary, no supplies available on the day and not being part of the priority group on the day); and whether they ever attempted to get vaccinated but were unsuccessful.

Regarding the variables, the dependent variable was having a complete vaccination status for each vaccine while the independent variables included gender, age (in years), education level, marital status and duration of diabetes (in years). Individuals were considered vaccinated against influenza if there was a record of one dose of the vaccine within the year prior to data collection; against hepatitis B, a record of three doses; for the MMR vaccine, two doses (for ages 20 to 29) and one dose (for ages 30 to 59); for yellow fever, a record of a single dose; for dT, three doses received within 10 years prior to data collection; for pneumococcus (PCV13) and varicella, two doses; and for COVID-19, two doses plus one booster.

### Data processing and analysis

The data were tabulated and organized in a Microsoft Excel^®^ 2016 spreadsheet and analyzed by using the Statistical Package for the Social Sciences (SPSS) version 26. For exploratory, descriptive analyses, absolute and relative frequencies were applied for qualitative variables, and measures of central tendency and dispersion for quantitative variables. The confidence interval was applied to the relative frequency and mean of the analyzed variables. To verify the factors associated with vaccination status, bivariate analysis was conducted using Fisher’s exact test (X²). For variables that showed significance at the 5% level, bivariate logistic regression was applied to calculate the odds ratio (OR). A significance level of 5% (0.05) was adopted, considering p-values < 0.05 as statistically significant for association.

### Ethical aspects

The research was approved by the Research Ethics Committee of the proposing institution under number 5,036,594, on October 14, 2021, and was conducted in accordance with Resolution 466/12 of the National Health Council.

## Results

Of the 274 individuals with DM who made up the sample, the majority were aged 60 or older (169; 62.6%), were women (192; 70.1%), had an income lower than or equal to one minimum wage (150; 63.6%) and had complete elementary education (139; 51.5%). Regarding clinical characteristics, the most reported type of DM was type 2 (260; 95.2%), with over 10 years of duration of the chronic condition (109; 41.3%), and treated with oral antidiabetic medication (231; 85.9%), as presented in Table 1.

Table 2 shows that the majority had a vaccination card (262; 95.6%), with no doses for hepatitis B (188; 69.1%); and the schedule for the influenza vaccine, 204 (75%) had received the single dose; most were not vaccinated against diphtheria and tetanus (175; 64.6%); yellow fever (202; 74.3%); MMP (223; 82.3%); pneumococcus (240; 87.9%); and varicella (239; 87.9%). Most had a complete vaccination schedule for COVID-19 (271; 68.5%). Regarding place of vaccination, the FHUs were the most frequently mentioned facilities (168; 62%).

**Table 1 -:** Sociodemographic and clinical profile of individuals with diabetes mellitus registered with the FHTs* (n = 274). Picos, PI, Brazil, 2022

Sociodemographic Profile	n(%)	95%CI^†^
Gender		
Women	192(70.1)	(64.5–75.3)
Men	82(29.9)	(24.7–35.5)
Age (years)		
20 to 59	101(37.4)	(31.8–43.3)
≥60	169(62.6)	(56.7–68.2)
Marital status		
Single/ Widowed	126(46.7)	(40.8–52.6)
Married/Cohabitation	144(53.3)	(47.4–59.2)
Educational level		
Illiterate	58(21.5)	(16.9–26.7)
Elementary	139(51.5)	(45.5–57.4)
Secondary	56(20.7)	(16.2–25.9)
Higher	15(5.6)	(3.3–8.8)
Literate	2(0.7)	(0.2–2.4)
Race/Skin color		
White	119(45.2)	(39.3–51.3)
Black	57(21.7)	(17.0–26.9)
Brown	86(32.7)	(27.2–38.5)
Other	1(0.4)	(0.0–1.8)
Religion		
Catholic	215(82.4)	(77.4–86.6)
Evangelical	35(13.4)	(9.7–17.9)
Other	11(4.2)	(2.3–7.2)
Income (minimum wages)^‡^		
≤1 MW	150(63.6)	(57.3–69.5)
>1 MW	86(36.4)	(30.5–42.7)
Clinical Profile		
Type of DM^§^		
Type 1	13(4.8)	(2.7–7.8)
Type 2	260(95.2)	(92.2–97.3)
Duration of DM^§^		
< 5 years	75(28.4)	(23.2–34.1)
5-10 years	80(30.3)	(25.0–36.0)
> 10 years	109(41.3)	(35.5–47.3)
Type of medication treatment		
Oral antidiabetics	231(85.9)	(81.3–89.6)
Insulin	24(8.9)	(6.0–12.8)
Both	14(5.2)	(3.0–8.4)
Type of non-medication treatment		
Physical exercise	31(11.4)	(8.0–15.5)
Nutritional education	81(29.7)	(24.5–35.3)
Both	54(19.8)	(15.4–24.8)
None	107(39.2)	(33.5–45.1)
Medication		
Always	253(97.3)	(94.8–98.8)
Rarely	4(1.5)	(0.5–3.6)
Only with symptoms	3(1.2)	(0.3–3.0)
Comorbidities		
Yes	49(17.9)	(13.7–22.7)
No	225(82.1)	(77.3–86.3)
Smoking		
Yes	28(10.2)	(7.1–14.2)
No	222(81.0)	(76.1–85.3)
Previously	24(8.8)	(5.8–12.5)
Alcohol consumption		
Yes	28(10.2)	(7.1–14.2)
No	200(73.0)	(67.5–78.0)
Previously, but no more	46(16.8)	(12.7–21.6)
Regular physical exercise (+ 3 times a week)		
Yes	71(25.9)	(21.0–31.3)
No	203(74.1)	(68.7–79.0)

*FHTs = Family Health Team; ^†^95%CI = Confidence Interval; ^‡^Minimum wage R$ 1,212.00, Brazil, 2022; ^§^DM = Diabetes Mellitus

**Table 2 -:** Vaccination status of individuals with diabetes mellitus registered with the FHTs* (n = 274). Picos, PI, Brazil, 2022

Vaccination Status	n (%)	95%CI^†^
Vaccination card		
Yes	262(95.6)	(92.7–97.6)
No	8(2.9)	(1.4–5.4)
Never had one	1(0.4)	(0.0–1.7)
Lost it	3(1.1)	(0.3–2.9)
Vaccination card shown		
Yes	215(78.5)	(73.3–83.0)
No	59(21.5)	(17.0–26.7)
If not, the vaccination status was:		
Self-reported	57(98.3)	(92.2–99.8)
Does not know	1(1.7)	(0.2–7.8)
Vaccination against hepatitis B		
Yes	58(21.3)	(16.8–26.5)
No	188(69.1)	(63.4–74.4)
Does not know	26(9.6)	(6.5–13.5)
Vaccination against influenza		
Yes	204(75.0)	(69.6–79.9)
No	64(23.5)	(18.8–28.8)
Does not know	4(1.5)	(0.5–3.5)
Vaccination against dT^‡^		
Yes	69(25.5)	(20.6–30.9)
No	175(64.6)	(58.8–70.1)
Does not know	27(10.0)	(6.8–14.0)
Vaccination against MMR		
Yes	18(6.6)	(4.1–10.1)
No	223(82.3)	(77.4–86.5)
Does not know	30(11.1)	(7.8–15.2)
Vaccination against yellow fever		
Yes	42(15.4)	(11.5–20.1)
No	202(74.3)	(68.8–79.2)
Does not know	28(10.3)	(7.1–14.3)
Vaccination against COVID-19		
Yes	271(99.3)	(97.7–99.8)
No	2(0.7)	(0.2–2.3)
Does not know	0(0.0)	
Reaction to this vaccine		
Yes	115(43.1)	(37.2–49.1)
No	152(56.9)	(50.9–62.8)
Vaccination against varicella		
Yes	4(1.5)	(0.5–3.5)
No	239(87.9)	(83.6–91.3)
Does not know	29(10.7)	(7.4–14.7)
Conjugate vaccination against pneumococcus (PCV10^§^, PCV13^||^)		
Yes	4(1.5)	(0.5–3.4)
No	240(87.9)	(83.7–91.4)
Does not know	29(10.6)	(7.4–14.7)
Place of vaccination		
FHU^¶^	168(62.0)	(56.1–67.6)
Home	2(0.7)	(0.2–2.3)
Secondary Care	101(37.3)	(31.7–43.1)
CRIE**	0(0.0)	

*FHT = Family Health Team; ^†^95%CI = Confidence Interval; ^‡^dT = Adult diphtheria and tetanus vaccine; ^§^PCV10 = 10-valent conjugate pneumococcal vaccine; ^||^PCV13 = 13-valent conjugate pneumococcal vaccine; ^¶^FHU = Family Health Unit; **CRIE = Special Immunobiology Referral Centers

Most of the respondents (165; 60.2%) reported never having heard of the PNI. In addition, nearly half (129; 48.3%) felt that the program’s media campaigns are not sufficient to motivate the public to get vaccinated. The main reasons mentioned for having an incomplete vaccination schedule were not knowing the importance of vaccination (68; 25.9%) and not being informed by the healthcare provider (98; 37.3%), as shown in Table 3.

**Table 3 -:** Perception about vaccination of individuals with diabetes mellitus registered with the FHTs* (n = 274). Picos, PI, Brazil, 2022

Perception about vaccination	n (%)	95%CI^†^
Knowledge about PNI^‡^		
Yes	109(39.8)	(34.1–45.7)
No	165(60.2)	(54.3–65.9)
Considers the PNI^‡^ media campaigns insufficient to encourage vaccination		
Yes	108(40.4)	(34.7–46.4)
No	129(48.3)	(42.4–54.3)
Partially	30(11.2)	(7.9–15.4)
Reason for incomplete vaccination status		
Fear of adverse reactions	21(8.0)	(5.2–11.7)
Not knowing the importance of vaccination	68(25.9)	(20.8–31.4)
Neglect	64(24.3)	(19.4–29.8)
Not considered necessary	7(2.7)	(1.2–5.2)
Not informed by healthcare provider	98(37.3)	(31.6–43.2)
Other	5(1.9)	(0.7–4.1)
Received guidance on the importance of being up to date with vaccines		
Yes	208(76.2)	(70.9–80.9)
No	65(23.8)	(19.1–29.1)
Follows recommendations by healthcare providers		
Yes	178(68.5)	(62.6–73.9)
No	46(17.7)	(13.4–22.7)
Partially	36(13.8)	(10.1–18.4)
Adverse effects after vaccination		
Yes	138(50.7)	(44.8–56.6)
No	134(49.3)	(43.4–55.2)
Returns to the FHU^§^ for vaccines that require additional doses		
Yes	220(83.0)	(78.2–87.2)
No	45(17.0)	(12.8–21.8)
Health problem that prevents taking a vaccine		
Yes	3(1.1)	(0.3–2.9)
No	271(98.9)	(97.1–99.7)
Medical/nursing recommendation not to take the vaccine following the health problem		
Yes	1(0.4)	(0.0–1.7)
No	272(99.6)	(98.3–100.0)
Difficulty to get vaccinated		
Yes	67(24.5)	(19.6–29.8)
No	207(75.5)	(70.2–80.4)
Any unsuccessful attempt to get vaccinated		
Yes	30(11.0)	(7.7–15.2)
No	242(89.0)	(84.8–92.3)

*FHT = Family Health Teams; ^†^95%CF = Confidence Interval; ^‡^PNI = National Immunization Program; ^§^FHU = Family Health Unit

**Figure 1 -:**
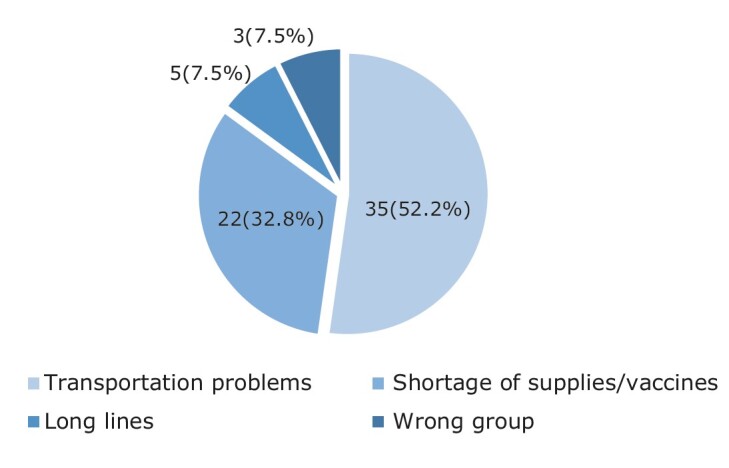
Main difficulties to get vaccinated faced by individuals with diabetes mellitus treated by the FHTs (n = 65). Picos, PI, Brazil, 2022

In this survey, the main difficulties reported by individuals with DM to get vaccinated include transportation problems (35; 52.2%) and shortage of supplies/vaccines (22; 32.8%), as shown in the Figure 1.

In Table 4, the odds ratios were analyzed among the variables that showed statistical significance (p<0.005) in the bivariate analysis. It was observed that vaccination against influenza was associated with age (OR = 0.395; CI 206–0.755); with being Catholic (OR = 6.275; CI 1.201–32.781) and evangelical (OR = 5.295; CI 0.878–31.950); with family income (OR = 0.321; CI 0.144–0.718); and with alcohol consumption (OR = 0.394; CI 0.148–1.050).

In the case of the MMR vaccine, type of DM did not influence the likelihood of a person with diabetes getting vaccinated (OR = 8.491), while those using oral antidiabetic medication have lower chances of being vaccinated compared to those using both treatments (insulin and oral antidiabetics) (OR = 0.313; CI 0.028–3.515). Regarding yellow fever vaccination, individuals with DM1 are more likely to get vaccinated compared to those with DM2 (OR = 4.286; CI 1.292–14.218).

Considering COVID-19 vaccination, younger individuals (aged 20 to 59) are less likely to be vaccinated compared to older individuals (60 years or older) (OR = 0.342; CI 0.153–0.761). People with DM1 are more likely to be vaccinated compared to those with DM2 (OR = 0.142; CI 0.029–0.753), and regarding the duration of diabetes, individuals with fewer years of the condition have lower chances of completing the vaccination schedule (OR = 0.132; CI 0.039–0.445) compared to those who have lived with the condition for a longer time (OR = 0.276; CI 0.078–0.968).

**Table 4 -:** Logistic regression analysis of the association between vaccination against influenza, dT*, MMR, yellow fever and COVID-19 and the sociodemographic and clinical profile of individuals with diabetes mellitus registered with the FHTs^†^ (n = 274). Picos, PI, Brazil, 2022

**Vaccine/Variable**	**OR** ^‡^	**(IC-95%)** ^§^	**p-value** ^||^
**Influenza**			
**Age (in years)**			
20 to 59	0.395	(0.206-0.755)	0.005
≥60			
**Religion**			
Catholic	6.275	(1.201-32.781)	0.029
Evangelical	5.295	(0.878-31.950)	0.069
Other			
**Household income (minimum wages)** ^¶^			
≤1 SM	0.321	(0.144-0.718)	0.006
>1 SM			
**Alcohol consumption**			
Yes	0.394	(0.148-1.050)	0.062
No	1.508	(0.728-3.124)	0.268
Previously, but no more			
dT*			
Duration of DM**			
< 5 years	3.429	(0,779-15,086)	0.103
5-10 years	0.429	(0,123-1,491)	0.183
> 10 years			
**Tríplice viral**			
**Education level**			
Illiterate	0.055	(0.003-1.102)	0.058
Elementary	0.030	(0.002-0.576)	0.020
Secondary	0.143	(0.008-2.552)	0.186
Higher	0.250	(0.012-5.262)	0.373
Type of DM**			
Type 1	8.491	(0.766-94.064)	0.081
Type 2			
**Medication treatment**			
Oral antidiabetics	0.187	(0.039-0.899)	0.036
Insuline	0.313	(0.028-3.515)	0.346
Both			
**Regular physical exercise (> 3 times/week)**			
Yes	0.108	(0.013-0.894)	0.108
No			
**Yellow Fever**			
Type of DM**			
Type 1	4.286	(1.292-14.218)	0.017
Type 2			
**COVID-19**			
**Age (in years)**			
20 to 59	0.342	(0.153-0.761)	0.09
≥60			
Type of DM**			
Type 1	0.142	(0.029-0.753)	0.021
Type 2			
Duration of DM**			
< 5 years	0.132	(0.039-0.445)	0.001
5-10 years	0.276	(0.078-0.968)	0.044
> 10 years			

*dT = Adult diphtheria and tetanus vaccine; ^†^FHTs = Family Health Teams; ^‡^OR = Odds ratio; ^§^95%CI = Confidence interval; ^||^p-value = Wald test at the 5% level; ^¶^Minimum wage R$ 1,212.00, Brazil, 2022; **DM = Diabetes mellitus

## Discussion

The results of this study indicate low vaccination rates regarding the list of recommended vaccines for individuals with DM. Most had taken no hepatitis B vaccine and only 21.3% presented a complete vaccination schedule (three doses), which is consistent with other studies assessing vaccination in individuals with DM^(10,12-14)^. This situation is concerning, as these individuals have high rates of hepatitis B^(4)^ and outbreaks of this disease have been associated with blood glucose monitoring procedures among people with DM^(16)^.

Regarding influenza vaccination, most participants had taken the single dose, which is administered annually. This finding may be related to the annual campaigns, media messages and the inclusion of DM in the group of chronic diseases prioritized for vaccination since 2013. Nevertheless, the goal of 90% vaccination coverage in Brazil was not achieved in 2022^(17)^.

In this study, it was identified that younger adults (aged 20 to 59) had a lower likelihood of vaccination compared to older individuals, which corroborates findings from other studies^(11-12,14)^ that reported a higher frequency of influenza vaccination in individuals over 65 and 60 years old, respectively. This is an essential prevention strategy for this age group, as the most frequent occurrences of SARS due to influenza in Brazil in 2021 were among individuals aged 60 and older, accounting for 52.1% (2,252) of the cases.

A retrospective cohort study in the United States found that individuals with influenza infection had significantly increased rates of pneumonia, ischemic heart disease and sepsis during the influenza period compared to baseline periods. For individuals with DM2, these results suggest that influenza increases the risk of more severe clinical outcomes and healthcare use compared to the general population^(18)^.

Most individuals with DM who took part in this survey also had low rates for the pneumococcal vaccine, similar to the finding of a study that evaluated this vaccine in individuals with DM receiving care in a referral healthcare facility in the Federal District. The study also examined the level of knowledge among healthcare professionals on the subject and the impact of guidance on vaccination adherence. It was found that only 12.1% of individuals with DM and pneumonia reported having received the pneumococcal vaccine, while 69.7% denied receiving it and 18.2% were unaware of its existence^(19)^.

Regarding the causes and weaknesses affecting pneumococcal vaccination coverage, it is observed that there is misinformation among prescribing professionals in APS about the recommendations of the Brazilian Ministry of Health (MS). In this context, some strategies are necessary such as improving the training of APS professionals on MS recommendations for adult vaccination, streamlining the request and dispensing of vaccines, simplifying information processes, encouraging multidisciplinary care and raising awareness among both professionals and patients about the importance of vaccination^(20)^.

It is important to note that this vaccine is available in the CRIEs upon medical prescription. Thus, two initiatives could enhance access and adherence to pneumococcal vaccination for individuals with DM: first, informing prescribing professionals about the importance of requesting this vaccine for individuals with DM from the CRIEs; and second, expanding the list of professionals who can request/prescribe the vaccine for individuals with DM.

A randomized clinical trial evaluated the vaccination rates of individuals with DM who received guidance on updating their vaccination schedules for influenza, hepatitis B, pneumonia and tetanus. No significant increase was observed, showing that these individuals did not receive adequate guidance from the professionals caring for them. It is crucial to offer training to healthcare professionals, raising their awareness about the importance of providing individuals with DM with guidance on vaccination during routine consultations^(21)^.

Concerning vaccination against COVID-19, the majority were up to date with their vaccination schedule. Young individuals who have lived with DM for less time and those with DM1 had a lower chance of being vaccinated compared to older adults who have lived with the condition for a longer time and those with DM2. Older adults have more health information, as they frequent health services more often than younger individuals. In addition, the COVID-19 vaccination campaigns stressed the importance of specific protection for high-risk groups due to the poor prognosis of the infection in older adults.

Individuals with DM are at greater risk for severe COVID-19 compared to those without DM. In such individuals, the chances of hospitalization due to COVID-19 and the greater severity of the disease are three to four times higher compared to those without DM^(22)^. Thus, primary prevention through timely vaccination remains essential to mitigate the risks associated with COVID-19 in people with DM.

The vaccination rate against diphtheria and tetanus was very low. It is known that individuals with DM2 are prone to tetanus infection, especially when diagnosed with “diabetic foot” lesions, because the reduced capillary circulation in the lower extremities leads to polymicrobial infections complicated by anaerobic bacteria^(8)^.

Regarding the varicella vaccine, despite being highly recommended by the SBD and the SBIm^(6)^, coverage rates were also negligible. For individuals with DM, this vaccine is available only at the CRIEs; therefore, it is necessary to raise awareness among prescribing professionals about its importance for this population. About 98% of Brazilian adults have a history of varicella by the age of 60, which may lead to the development of herpes zoster^(6)^. Consequently, these individuals have a substantially increased risk of developing herpes zoster, along with its complications such as pain from the acute phase and post-herpetic neuralgia^(24)^.

Regarding the perception of individuals with DM about vaccination, most had never heard of the PNI, which is recognized as one of the largest public programs for access to vaccination worldwide. In Brazil, it is publicized as an important health strategy; however, it could be more widely communicated among the Brazilian adult and older adult population, especially by professionals working in APS.

In this survey, one of the most significant reasons for having an incomplete immunization schedule was not being informed by healthcare providers about the importance of getting vaccinated. It is known that professional recommendation is a fundamental strategy in the decision to accept vaccination^(21,25)^. Health professionals should give clear and accessible information regarding how vaccination services operate, including their respective vaccination schedules and campaigns^(9)^.

The healthcare providers who most informed individuals with DM about the importance of vaccination were the ACS and nurses. It is noteworthy that these professionals have more frequent contact with people with DM and should take the opportunity to instruct them about the benefits of maintaining their vaccination schedules up to date.

Effective communication is crucial not only to convey information about the importance of vaccination as a preventive measure but also to boost people’s confidence in vaccines. In addition, the credibility of the PNI among the population should be reinforced^(26)^ to highlight the benefits of vaccination and dispel doubts that influence the decision to get vaccinated.

The reasons mentioned for incomplete immunization schedules include lack of understanding of the importance of vaccination and poor information provided by professionals. It is known that such agents facilitate vaccination adherence by encouraging individuals and reassuring them about safety and adverse effects^(9)^. Research^(26)^ has indicated that fear of adverse events compromises access to vaccination, in addition to other socioeconomic and geographic factors. Understanding these factors is crucial for planning vaccination strategies adapted to existing disparities and the need for more equitable policies in this context.

Therefore, it is essential to intensify campaigns, provide clarification and facilitate vaccination practices among patients, especially young adults, who are more reluctant to seek healthcare services. In this sense, nurses play an important role as health educators, providing communication channels to spread information about vaccination and boost adherence to it^(27)^.

For all vaccines, most of individuals with DM were vaccinated in healthcare facilities of the Unified Health System (SUS), especially the FHUs. This consolidates the PNI’s condition as a vital public health program for universal access, ensuring that essential vaccines are available to the entire population, regardless of socioeconomic or geographic condition. This is also linked to increasing population coverage through APS.

Among the obstacles to vaccination, the most frequently reported were difficulties getting to vaccination sites due to physical limitations or lack of transport, as well as shortages of supplies or vaccines at the time of immunization. Notably, this population is predominantly composed of older adults, many of whom face some degree of physical limitation due to aging and have low incomes, which hinders access to healthcare services.

In light of this, healthcare facilities, especially in APS, should implement strategies to reach individuals with access difficulties and coordinate home care services to ensure the provision of vaccination. An additional element identified as hindering access to vaccination was lack of supplies/vaccines; therefore, it is essential to plan for the required number of doses. Aspects related to the organization and planning of vaccination campaigns may influence patient adherence.

The limitations of this study pertain to the local context in which the survey was conducted, making it difficult to generalize the results to other regions. Therefore, further research is recommended to understand other settings, as well as the barriers related to vaccination practices in the population with DM.

Regarding advancements in scientific knowledge, this is the first nationwide study to analyze the immunization status of people with DM for the vaccines recommended by the PNI^(5-6)^, including those against COVID-19. In addition, it surveyed their perceptions regarding vaccination and the difficulties faced in primary healthcare.

Therefore, this study raised reflections on the importance of vaccination among these individuals, considering the encouragement of healthcare professionals, especially nurses, to provide guidance on the relevance of vaccination and to create opportune moments for vaccination, such as during visits for procedures like capillary blood glucose testing. Furthermore, the study is expected to help address the causes of low vaccination rates, such as poor professional guidance. This includes adopting and strengthening practices like filling out vaccination cards for individuals with DM and using the eSUS-APS system to record administered doses.

## Conclusion

It was found that individuals with DM 1 and 2 treated in primary healthcare have low vaccination rates regarding the vaccines recommended by the PNI for this population group. This is concerning, as these individuals are more vulnerable to vaccine-preventable infections and mortality, which can significantly worsen their quality of life if they develop a preventable disease.

Efforts should be made to understand the barriers to vaccination and enhance the vaccination status of individuals with DM through ongoing training of healthcare providers and increased public awareness. The role of the nursing team is crucial in implementing vaccination initiatives across the country, as their involvement can significantly improve the vaccination rates among individuals with diabetes mellitus.
