# Supplementary material for: Vaccination status of individuals with diabetes mellitus treated in Primary Healthcare: a cross-sectional study
Source: Rev Lat Am Enfermagem. 2025 Feb 3;33:e4452. doi: 10.1590/1518-8345.7065.4452 (PMC11789757; doi:10.1590/1518-8345.7065.4452)
Supplement: Translation

# Translation: Situación vacunal de personas con diabetes mellitus asistidas en la Atención Primaria de Salud: estudio transversal*

**DOI:** 10.1590/1518-8345.7065.4451

**Authors:** Francisco João de Carvalho, Luisa Helena de Oliveira Lima, Mônica Antar Gamba, Rosilane Lima de Brito, Lucilane Maria Sales da Silva, Ana Roberta Vilarouca da Silva

**Affiliations:** 1 Universidade Estadual do Ceará, Fortaleza, CE, Brasil.; 2 Becario de la Fundação Cearense de Apoio ao Desenvolvimento Científico e Tecnológico (FUNCAP), Brasil.; 3 Universidade Federal do Piauí, Teresina, PI, Brasil.; 4 Universidade Federal de São Paulo, São Paulo, SP, Brasil.

**Keywords:** Cobertura de Vacunación, Diabetes Mellitus Tipo 2, Diabetes Mellitus Tipo 1, Atención Primaria de Salud, Enfermería, Vacunas

## Abstract

analizar la situación vacunal de personas con diabetes mellitus tipos 1 y 2 en la atención primaria de salud.

estudio transversal, analítico, realizado en 25 equipos de Salud de la Familia con 274 personas con diabetes. Se evaluaron las variables sociodemográficas, clínicas y el esquema vacunal completo para cada vacuna recomendada por el Programa Nacional de Inmunizaciones mediante un análisis bivariado y regresión logística.

de las personas con diabetes mellitus, el 69,1% no contaba con el esquema vacunal completo contra la hepatitis B; el 64,6% contra la difteria y el tétanos; el 74,3% contra la fiebre amarilla; el 87,9% contra el neumococo; el 87,9% contra la varicela; el 24,5% contra influenza y el 0,7% contra la COVID-19. Entre los motivos aludidos para el esquema vacunal incompleto, figuran no conocer la importancia de vacunarse y no haber sido informado por el profesional de la salud. Hubo asociación estáticamente significante entre el perfil sociodemográfico y clínico con relación al esquema vacunal completo entre la vacuna contra la influenza y la edad y los ingresos; COVID-19 y edad, tipo de diabetes y tiempo viviendo con diabetes.

las personas con diabetes mellitus tipos 1 y 2 presentaron baja completitud de las vacunas en el contexto de la Atención Primaria de Salud, lo que es preocupante debido a la situación de mayor vulnerabilidad a infecciones inmunoprevenibles y mortalidad en este grupo.

## Destacados

**(1)** Las personas con diabetes presentan baja completitud de las vacunas.

**(2)** Es fundamental brindar oportunidades de vacunación en los servicios de salud a las personas con diabetes

**(3)** Es necesaria la educación de los profesionales que prescriben vacunas.

**(4)** Los jóvenes tienen menos probabilidades de vacunarse que las personas mayores.

**(5)** Es fundamental conocer los factores que predisponen a la situación vacunal.

## Introducción

Se estima que 537 millones de adultos de entre 20 y 79 años en todo el mundo tienen diabetes mellitus (DM). Para el 2030, la estimación es de 643 millones, y para el 2045, de 783 millones. En el escenario brasileño, el país está entre los 10 primeros territorios en número de adultos (20 a 79 años) con DM en el 2021, ocupando la 6ª posición^(1)^.

Esa condición de hiperglucemia persistente interfiere significativamente en la eficacia del sistema inmunológico de estas personas, ya que se produce compromiso de los mecanismos de defensa, que incluyen la supresión de la producción de citoquinas, defectos en la fagocitosis, disfunción de las células inmunológicas e incapacidad para detener a los microorganismos^(2-3)^. Esto las hace más susceptibles a ciertas enfermedades infecciosas^(3)^, que podrían prevenirse con la vacunación, lo que tendría impactos en la reducción de la morbilidad y hospitalizaciones^(4)^.

La vacunación de personas con DM es una estrategia fundamental para proteger la salud y promover la calidad de vida. Cabe resaltar que estas deben recibir atención especializada, ya que tienen probabilidades aumentadas en: 2,5 veces para desarrollar el Síndrome Respiratorio Agudo Severo (SARS) por influenza; 1,4 veces para la neumonía adquirida en la comunidad; 4,6 veces para la enfermedad neumocócica invasiva; dos veces por complicaciones hepáticas cuando también tienen hepatitis B crónica, y tres veces por herpes zóster^(5)^.

En consonancia con las Sociedades Brasileñas de Diabetes (SBD) e Inmunización (SBIm), y teniendo en cuenta las recomendaciones de los Centros de Referencia de Inmunobiológicos Especiales (CRIE), las siguientes vacunas son sumamente recomendadas para personas con diabetes: influenza, neumocócica conjugada 13-valente (VPC13), neumocócica polisacárida 23-valente (VPP23), hepatitis B, *Haemophilus influenzae* tipo b (Hib), varicela y herpes zóster, resaltando que la mayoría está disponible en el Programa Nacional de Inmunizaciones (PNI)^(6)^.

Entre los inmunobiológicos disponibles en la Atención Primaria de Salud (APS) y en los CRIE se encuentran las vacunas contra la influenza, la hepatitis B, la fiebre amarilla, la difteria y el tétanos adulto (dT), la triple vírica y COVID-19. Las vacunas neumocócicas VPP23, conjugada 13-valente VPC13 y varicela, aunque recomendadas, no están disponibles en la APS para personas con DM, solo en los CRIE bajo prescripción médica. Y la vacuna contra el herpes zóster solo está disponible en la red privada.

La vacunación es un pilar esencial de la APS y representa un derecho humano incuestionable. En las unidades de la red de APS se realiza la verificación de la libreta y de la situación vacunal, además de la orientación y administración para iniciar o completar el esquema vacunal, según los calendarios específicos^(7)^. Además, el registro detallado de las vacunas recibidas por las personas con DM, junto con la evaluación de sus necesidades, constituyen elementos esenciales de una evaluación clínica holística, realizada al inicio del seguimiento y durante las consultas anuales^(7)^.

Los determinantes sociales influyen en la decisión de vacunarse. Entre los factores socioeconómicos y clínicos asociados con la condición de estar vacunado, repercuten negativamente en la vacunación menor educación e ingresos, mientras que la edad avanzada se asocia con tasas de vacunación más altas^(8)^. Además, los factores logísticos y relacionados con los profesionales de la salud pueden suponer barreras para la vacunación^(9)^.

Sin embargo, a pesar de las recomendaciones, los estudios nacionales^(10-11)^ e internacionales^(12-14)^ que evaluaron la vacunación en personas con DM no incluyeron todas las vacunas recomendadas para este grupo. Además de llenar este vacío, la presente investigación también aborda la percepción de las personas con DM sobre la vacunación, así como las respectivas dificultades que enfrentan para vacunarse. Ante ello, la planificación y la implementación de estrategias para llegar a la población con DM son imprescindibles y urgentes. Por tanto, el objetivo fue analizar la situación vacunal de las personas con diabetes mellitus tipos 1 y 2 en la Atención Primaria de Salud.

## Método

### Tipo de estudio

Estudio transversal, analítico, basado en las directrices del *Strengthening the Reporting of Observational Studies in Epidemiology* (STROBE)^(15)^.

### Lugar

La investigación se desarrolló en un municipio de Piauí, Brasil, ubicado en el centro-sur del estado, con una población estimada de 83.000 habitantes. Tiene una cobertura del 100% en APS, con 36 equipos de Salud de la Familia (eSF), 25 de los cuales ubicados en zonas urbanas y 11 en zonas rurales. Estos equipos conforman las Unidades de Salud de la Familia (USF), cuyos establecimientos cuentan con al menos una eSF.

### Período

La recopilación de datos tuvo lugar de enero a julio del 2022.

### Población y muestra

La población estuvo conformada por los usuarios registrados en las 25 eSF ubicadas en la zona urbana del municipio estudiado. El sistema de información utilizado para el registro de personas con DM es el e-SUS APS. Se utilizó el cálculo de muestras para una población finita, estratificada por proporción, totalizando 2.564 personas con DM tipo 1 (DM1) y DM tipo 2 (DM2), con un error de muestreo del 5% y un nivel de confianza del 95%. Para efectos de prevalencia, se utilizó 0,5 con el objetivo de obtener la muestra máxima, considerando un nivel de confianza del 95% y de significancia del 5%. Una ventaja que permitió el muestreo estratificado proporcional es la distribución proporcional de las personas con DM registradas, según el tamaño poblacional de cada eSF. Así, la muestra final se constituyó de 274 personas con DM.

### Criterios de selección

Se incluyeron personas con 18 años de edad o más, diagnosticadas con DM1 o DM2, registradas en la USF de alcance desde hace al menos un año. Se excluyeron aquellas que presentaron algún déficit cognitivo que imposibilitara la respuesta directa a las preguntas del instrumento de recopilación de datos evaluado mediante la aplicación del Miniexamen del Estado Mental.

### Recopilación de datos

Esta etapa se realizó mediante la aplicación del formulario de recopilación de datos durante las consultas de enfermería a las personas con DM que aceptaron participar en la investigación, mediante la firma del Consentimiento Libre, Previo e Informado (CLPI). En el caso de las personas que no asistían a la USF, se realizaron visitas domiciliarias, acompañadas de agentes comunitarios de salud (ACS). El proceso siguió los pasos a continuación: 1- Celebración de reuniones con los ACS y enfermeros, incluido el sorteo de los participantes con base en la información disponible sobre personas con DM en cada eSF; 2- Solicitud del número de teléfono de la persona sorteada para conformar la muestra; 3- Programación de la visita a la residencia mediante contacto telefónico previo, en el que se explicaron el CLPI y las condiciones de ingreso al domicilio; 4- A quienes aceptaron participar se les solicitó un espacio adecuado en el domicilio para la aplicación del instrumento de recopilación de datos; 5- Se utilizaron las condiciones de ingreso al domicilio para acceder a la residencia. Debido a la pandemia de COVID-19, se implementaron medidas de protección para garantizar la seguridad tanto de los investigadores como de los entrevistados.

### Instrumento de recopilación y variables del estudio

El investigador elaboró un instrumento de recopilación de datos titulado “Situación vacunal de personas con diabetes en la APS”, basado en las recomendaciones de SBD y SBIm y una revisión de la literatura sobre el tema. Con 30 ítems, con preguntas de opción múltiple y abiertas, divididos en tres dimensiones, a saber: caracterización sociodemográfica y clínica (16 ítems), que abarca variables como sexo, educación, ingresos, color, religión, ocupación, tipo de DM, tiempo viviendo con DM en años, tratamiento farmacológico y no farmacológico, comorbilidades, si tomaba siempre la medicación, si tenía hábitos de fumar y beber y si practicaba ejercicio físico regular (más de tres veces por semana).

La dimensión acerca de la situación vacunal (tres ítems) evalúa si la persona tiene libreta de vacunación, si ha verificado el esquema vacunal completo contra hepatitis b, influenza, dT, triple vírica, fiebre amarilla, COVID-19, varicela y neumococo conjugado y el lugar donde recibió la vacuna (USF, atención secundaria, domicilio o CRIE).

La última dimensión se refiere a la percepción de las personas con DM sobre la vacunación (11 ítems) que incluyó: conocimiento sobre el PNI; consideran suficientes las campañas mediáticas realizadas por el PNI para motivar la vacunación; razón por el cual el esquema vacunal estaba incompleto (miedo a experimentar reacciones adversas, desconocimiento sobre la importancia de vacunarse, experiencia previa de reacciones vacunales a causa de una vacuna, no considerarla necesaria, falta de información por parte del profesional de salud, otros motivos: especificarlos). Los participantes podían responder a más de un ítem. Además, se cuestionaba sobre la orientación por parte de un profesional de la salud acerca de la importancia de mantener actualizado el esquema vacunal; cumplimiento de las pautas de vacunación que brindaron los profesionales de salud; regreso a la USF para completar el esquema de las vacunas con dos o más dosis; efecto adverso posterior a la vacuna; problema de salud que impidió tomar la vacuna; orientación médica/de enfermería de no volver a vacunarse después de este problema de salud; dificultades para vacunarse (USF muy lejos, poco tiempo para ir, horario de atención de la clínica inadecuado, le dijeron que regresara otro día, no lo consideró necesario, cuando llegó no había insumos, no estaba en el grupo prioritario ese día); y si alguna vez fue a vacunarse y no lo logró.

En cuanto a las variables, la dependiente fue la situación vacunal completa de cada vacuna y las independientes fueron sexo, edad (en años), educación, situación conyugal, tiempo de diabetes (años). Se consideró vacunadas contra la influenza a las personas que presentaron un registro de una dosis de la vacuna hasta un año antes de su recopilación; contra la hepatitis B, registro de tres dosis; de la triple vírica, dos dosis (20 a 29 años) y una dosis (30 a 59 años); fiebre amarilla, registro de dosis única; dT tres dosis tomadas dentro de los 10 años anteriores a la recopilación; neumocócica conjugada (VPC13); varicela, dos dosis; COVID-19, dos dosis y un refuerzo.

### Tratamiento y análisis de datos

Los datos se tabularon y se organizaron en una hoja de cálculo electrónica *Microsoft Excel* ^®^ 2016 y se analizaron utilizando el *software Statistical Package for the Social Sciences* (SPSS) versión 26. Para los análisis descriptivos exploratorios, se aplicaron la frecuencia absoluta y relativa para las variables cualitativas y las medidas de posición y dispersión para las variables cuantitativas. Se aplicó el intervalo de confianza para la frecuencia relativa y media de las variables analizadas. Para verificar los factores asociados a la condición de estar vacunado, se realizó un análisis bivariado mediante la prueba exacta de Fisher (X^2^). Para las variables que presentaron significancia al nivel del 5%, se aplicó la regresión logística bivariada para calcular la razón de probabilidades o *Odds Ratio* (OR). Se adoptó un nivel de significancia del 5% (0,05), considerando valores de p < 0,05 como estadísticamente significativos para la asociación.

### Aspectos éticos

La investigación fue aprobada por el Comité de Ética en Investigación de la institución proponente bajo el número 5.036.594, el 14 de octubre del 2021, por lo que se realizó en consonancia con la resolución 466/12 del Consejo Nacional de Salud.

## Resultados

De las 274 personas con DM que integraron la muestra, la mayoría tenía 60 años o más (169; 62,6%), era mujer (192; 70,1%), con ingresos inferiores o iguales a un salario mínimo (150; 63,6%), educación primaria (139; 51,5%). En cuanto a las características clínicas, el tipo de DM más reportado fue la DM2 (260; 95,2%), vivir con la condición crónica por más de 10 años (109; 41,3%), utilizar antidiabéticos orales como tratamiento farmacológico (231; 85,9%), como se presenta en la Tabla 1.

La Tabla 2 muestra que la mayoría contaba con libreta de vacunación (262; 95,6%), sin ninguna dosis de hepatitis B (188; 69,1%); y con calendario para la vacuna contra la influenza, 204 (75%) tenía una sola dosis; la mayoría no contaba con calendario de vacunación contra: difteria y tétanos (175; 64,6%); fiebre amarilla (202; 74,3%); triple vírica (223; 82,3%), neumocócica (240; 87,9%) y varicela (239; 87,9%). La mayor parte presentó esquema vacunal completo para COVID-19 (271; 68,5%). En cuanto al lugar en el que se tomó la vacuna, la USF fue la más citada (168; 62%).

**Tabla 1 -:** Caracterización del perfil sociodemográfico y clínico de las personas con diabetes mellitus registradas en la eSF* (n = 274). Picos, PI, Brasil, 2022

Perfil Sociodemográfico	n(%)	IC-95%^†^
Sexo		
Femenino	192(70,1)	(64,5- 75,3)
Masculino	82(29,9)	(24,7- 35,5)
Rango de edad		
20 a 59 años	101(37,4)	(31,8- 43,3)
≥60 anos	169(62,6)	(56,7- 68,2)
Situación conyugal		
Soltero(a)/Viudo(a)	126(46,7)	(40,8-52,6)
Casado(a)/Unión estable	144(53,3)	(47,4- 59,2)
Escolaridad		
Analfabeto(a)	58(21,5)	(16,9-26,7)
Educación primaria	139(51,5)	(45,5-57,4)
Educación secundaria	56(20,7)	(16,2-25,9)
Educación superior	15(5,6)	(3,3-8,8)
Alfabetización	2(0,7)	(0,2-2,4)
Raza/color de piel		
Blanco	119(45,2)	(39,3- 51,3)
Negro	57(21,7)	(17,0- 26,9)
Pardo	86(32,7)	(27,2-38,5)
Otros	1(0,4)	(0,0-1,8)
Religión		
Católico(a)	215(82,4)	(77,4-86,6)
Evangélico(a)	35(13,4)	(9,7-17,9)
Otra	11(4,2)	(2,3-7,2)
Ingresos (salarios mínimos)^‡^		
≤1 SM	150(63,6)	(57,3-69,5)
>1 SM	86(36,4)	(30,5-42,7)
Perfil clínico		
Tipo de DM^§^		
Tipo 1	13(4,8)	(2,7-7,8)
Tipo 2	260(95,2)	(92,2-97,3)
Tiempo viviendo con DM^§^		
< 5 años	75(28,4)	(23,2-34,1)
5-10 años	80(30,3)	(25,0-36,0)
> 10 años	109(41,3)	(35,5-47,3)
Tipo de tratamiento farmacológico		
Antidiabéticos orales	231(85,9)	(81,3-89,6)
Insulina	24(8,9)	(6,0-12,8)
Ambos	14(5,2)	(3,0-8,4)
Tipo de tratamiento no farmacológico		
Ejercicios físicos	31(11,4)	(8,0-15,5)
Educación alimentaria	81(29,7)	(24,5-35,3)
Ejercicios físicos y educación alimentaria	54(19,8)	(15,4-24,8)
Ninguno	107(39,2)	(33,5-45,1)
Medicación		
Siempre	253(97,3)	(94,8-98,8)
Rara vez	4(1,5)	(0,5-3,6)
Solo con síntomas	3(1,2)	(0,3-3,0)
Comorbilidades		
No	49(17,9)	(13,7-22,7)
Sí	225(82,1)	(77,3-86,3)
Tabaquismo		
Sí	28(10,2)	(7,1-14,2)
No	222(81,0)	(76,1-85,3)
Ya fui fumador	24(8,8)	(5,8-12,5)
Etilismo		
Sí	28(10,2)	(7,1- 14,2)
No	200(73,0)	(67,5-78,0)
Solía consumir alcohol, pero ya no lo consumo	46(16,8)	(12,7-21,6)
Ejercicio físico regular (más de 3 veces por semana)		
Sí	71(25,9)	(21,0-31,3)
No	203(74,1)	(68,7-79,0)

*eSF = Equipo de Salud de la Familia; ^†^IC-95% = Intervalo de Confianza; ^‡^Salario mínimo R$ 1.212,00, Brasil, 2022; ^§^DM = Diabetes mellitus

**Tabla 2 -:** Situación vacunal de las personas con diabetes mellitus registradas en las eSF* (n = 274). Picos, PI, Brasil, 2022

Situación vacunal	n (%)	IC-95%^†^
Tiene libreta de vacunación		
Sí	262(95,6)	(92,7- 97,6)
No	8(2,9)	(1,4-5,4)
Nunca la tuve	1(0,4)	(0,0-1,7)
La tuve una vez, pero la perdí	3(1,1)	(0,3-2,9)
Libreta de vacunación vista		
Sí	215(78,5)	(73,3-83,0)
No	59(21,5)	(17,0-26,7)
En caso contrario, el esquema vacunal fue:		
Autorreferido	57(98,3)	(92,2-99,8)
No lo sabía	1(1,7)	(0,2-7,8)
Tiene un esquema vacunal contra la hepatitis B		
Sí	58(21,3)	(16,8-26,5)
No	188(69,1)	(63,4-74,4)
No lo sé	26(9,6)	(6,5-13,5)
Tiene un esquema vacunal contra la influenza		
Sí	204(75,0)	(69,6-79,9)
No	64(23,5)	(18,8-28,8)
No lo sé	4(1,5)	(0,5-3,5)
Tiene calendario de vacunación para dT^‡^		
Sí	69(25,5)	(20,6- 30,9)
No	175(64,6)	(58,8-70,1)
No lo sé	27(10,0)	(6,8-14,0)
Tiene calendario de vacunación de la triple vírica		
Sí	18(6,6)	(4,1- 10,1)
No	223(82,3)	(77,4-86,5)
No lo sé	30(11,1)	(7,8-15,2)
Tiene esquema vacunal contra la fiebre amarilla		
Sí	42(15,4)	(11,5- 20,1)
No	202(74,3)	(68,8-79,2)
No lo sé	28(10,3)	(7,1-14,3)
Tiene calendario de vacunación contra COVID-19		
Sí	271(99,3)	(97,7-99,8)
No	2(0,7)	(0,2-2,3)
No lo sé	0(0,0)	
Tuvo reacciones adversas a esta vacuna		
Sí	115(43,1)	(37,2- 49,1)
No	152(56,9)	(50,9-62,8)
Tiene un calendario de vacunación contra la varicela		
Sí	4(1,5)	(0,5-3,5)
No	239(87,9)	(83,6-91,3)
No lo sé	29(10,7)	(7,4-14,7)
Cuenta con un esquema vacunal contra la bacteria neumocócica conjugada (VPC10^§^, VPC13^||^)		
Sí	4(1,5)	(0,5-3,4)
No	240(87,9)	(83,7-91,4)
No lo sé	29(10,6)	(7,4-14,7)
Lugar en que recibió la vacuna		
USF^¶^	168(62,0)	(56,1-67,6)
Domicilio	2(0,7)	(0,2-2,3)
Atención secundaria	101(37,3)	(31,7-43,1)
CRIE**	0(0,0)	

*eSF = Equipo de Salud de la Familia; ^†^IC-95% = Intervalo de Confianza; ^‡^dT = Vacuna doble adulto; ^§^VPC10 = Vacuna neumocócica conjugada 10-valente; ^||^VPC13 = Vacuna neumocócica conjugada 13-valente; ^¶^USF = Unidad de Salud de la Familia; **CRIE = Centro de Referencia de Inmunobiológicos Especiales

La mayoría de los entrevistados (165; 60,2%) afirmó nunca haber oído hablar del PNI. Además, casi la mitad (129; 48,3%) consideró que las campañas que realiza el programa a través de los medios de comunicación no son suficientes para motivar al público a vacunarse. Los principales motivos mencionados para la incompletitud del calendario de vacunación fueron no conocer la importancia de la vacunación (68; 25,9%) y no haber sido informado por el profesional de salud (98; 37,3%), como se muestra en la Tabla 3.

**Tabla 3 -:** Percepción de las personas con diabetes mellitus registradas en las eSF* sobre vacunación (n = 274). Picos, PI, Brasil, 2022

Percepción sobre la vacunación	n (%)	IC-95%^†^
Conocimiento del PNI^‡^		
Sí	109(39,8)	(34,1- 45,7)
No	165(60,2)	(54,3-65,9)
Considera las campañas realizadas por el PNI^‡^, a través de los medios de comunicación, suficientes para motivar la vacunación		
Sí	108(40,4)	(34,7-46,4)
No	129(48,3)	(42,4-54,3)
Parcialmente	30(11,2)	(7,9-15,4)
Motivo del esquema vacunal incompleto		
Miedo a las reacciones adversas.	21(8,0)	(5,2-11,7)
No conoce la importancia de la vacunación	68(25,9)	(20,8-31,4)
Descuido	64(24,3)	(19,4-29,8)
No cree que lo necesita	7(2,7)	(1,2-5,2)
No fue informado por el profesional de salud	98(37,3)	(31,6-43,2)
Otros	5(1,9)	(0,7-4,1)
Recibió orientación acerca de la importancia de mantener actualizado el esquema vacunal		
Sí	208(76,2)	(70,9-80,9)
No	65(23,8)	(19,1-29,1)
Sigue las orientaciones de vacunación proporcionadas por profesionales de salud		
Sí	178(68,5)	(62,6-73,9)
No	46(17,7)	(13,4-22,7)
Parcialmente	36(13,8)	(10,1-18,4)
Efecto adverso tras la vacunación		
Sí	138(50,7)	(44,8-56,6)
No	134(49,3)	(43,4-55,2)
Para las vacunas que requieren regresar a la USF^§^ para completar el calendario		
Sí	220(83,0)	(78,2- 87,2)
No	45(17,0)	(12,8- 21,8)
Tiene un problema de salud que le impide vacunarse		
Sí	3(1,1)	(0,3-2,9)
No	271(98,9)	(97,1-99,7)
Orientación (médica/enfermería) de no vacunarse después de ese problema de salud		
Sí	1(0,4)	(0,0- 1,7)
No	272(99,6)	(98,3-100,0)
Dificultad para vacunarse		
Sí	67(24,5)	(19,6- 29,8)
No	207(75,5)	(70,2-80,4)
¿Alguna vez quiso vacunarse y no lo logró?		
Sí	30(11,0)	(7,7- 15,2)
No	242(89,0)	(84,8- 92,3)

*eSF = Equipos de Salud de la Familia; ^†^IC-95% = Intervalo de Confianza; ^‡^PNI = Programa Nacional de Inmunizaciones; ^§^USF = Unidad de Salud de la Familia

En esta investigación, las principales dificultades reportadas por las personas con DM para la vacunación incluyen la dificultad para desplazarse (35; 52,2%) y la escasez de insumos/vacunas (22; 32,8%), como muestra la Figura 1.

**Figura 1 -:** Principales dificultades para vacunarse de las personas con diabetes mellitus atendidas en la eSF (n = 65). Picos, PI, Brasil, 2022

En la Tabla 4, se analizó la razón de probabilidad entre las variables que presentaron significación estadística (p<0,005) en el análisis bivariado. Así, se observó que la vacunación contra la influenza se asoció con la edad (OR=0,395; IC206-0,755); con la religión católica (OR=6,275; IC1,201-32,781) y evangélica (OR= 5,295; IC0,878-31,950); con los ingresos familiares (OR=0,321; IC0,144-0,718) y con el consumo de alcohol (OR=0,394; IC0,148-1,050).

En el caso de la vacuna triple vírica, el tipo de DM no influyó en la probabilidad de que una persona con DM se vacunara (0R=8,491), mientras que quienes utilizan antidiabéticos orales tienen menos posibilidades de vacunarse en comparación con quienes utilizan ambos tratamientos (insulina y antidiabéticos orales) (OR=0,313; IC0,028-3,515). En cuanto a la fiebre amarilla, las personas con DM1 tienen más probabilidades de vacunarse en comparación con las que tienen DM2 (OR=4,286; IC1,292-14,218).

Con respecto a la vacunación contra COVID-19, las personas más jóvenes (20 a 59 años) tienen menos probabilidades de tener un esquema vacunal completo contra COVID-19 en comparación con las personas mayores (60 años o más) (OR=0,342; IC0,153-0,761), las personas con DM1 tuvieron más propensión a vacunarse en comparación con aquellas con DM2 (OR=0,142; IC0,029-0,753) y en cuanto al tiempo de vida con DM, las personas con menos años tienen menos probabilidades de completar el calendario de vacunación (OR=0,132; IC0,039-0,445) con relación a quienes han vivido más tiempo con la condición (OR=0,276; IC0,078-0,968).

**Tabla 4 -:** Análisis de regresión logística de la asociación entre el calendario de vacunación contra influenza, dT*, triple vírica, fiebre amarilla y COVID-19 y perfil sociodemográfico y clínico de las personas con diabetes mellitus registradas en el eSF^†^ (n = 274). Picos, PI, Brasil, 2022

**Vacuna/Variable**	**OR** ^‡^	**(IC-95%)** ^§^	**p-valor** ^||^
**Influenza**			
**Rango de edad**			
20 a 59 años	0,395	(0,206-0,755)	0,005
≥60 años			
**Religión**			
Católico(a)	6,275	(1,201-32,781)	0,029
Evangélico(a)	5,295	(0,878-31,950)	0,069
Otra			
**Ingreso familiar (salarios mínimos)** ^¶^			
≤1 SM	0,321	(0,144-0,718)	0,006
>1 SM			
**Etilismo**			
Sí	0,394	(0,148-1,050)	0,062
No	1,508	(0,728-3,124)	0,268
Solía consumir alcohol, pero ya no lo consumo			
dT*			
Tiempo viviendo con DM**			
< 5 años	3,429	(0,779-15,086)	0,103
5-10 años	0,429	(0,123-1,491)	0,183
> 10 años			
**Triple vírica**			
**Escolaridad**			
Analfabeto(a)	0,055	(0,003-1,102)	0,058
Educación primaria	0,030	(0,002-0,576)	0,020
Educación secundaria	0,143	(0,008-2,552)	0,186
Educación superior	0,250	(0,012-5,262)	0,373
Tipo de DM**			
Tipo 1	8,491	(0,766-94,064)	0,081
Tipo 2			
**Tratamiento farmacológico**			
Antidiabéticos orales	0,187	(0,039-0,899)	0,036
Terapia con insulina	0,313	(0,028-3,515)	0,346
Ambos			
**Ejercicio físico regular (> 3 veces/semana)**			
Sí	0,108	(0,013-0,894)	0,108
No			
**Fiebre amarilla**			
Tipo de DM**			
Tipo 1	4,286	(1,292-14,218)	0,017
Tipo 2			
**COVID-19**			
**Rango de edad**			
20 a 59 años	0,342	(0,153-0,761)	0,09
≥60 años			
Tipo de DM**			
Tipo 1	0,142	(0,029-0,753)	0,021
Tipo 2			
Tiempo viviendo con DM**			
< 5 años	0,132	(0,039-0,445)	0,001
5-10 años	0,276	(0,078-0,968)	0,044
> 10 años			

*dT = Doble vacuna para adultos; ^†^eSF = Equipos de Salud de la Familia; ^‡^OR = Regresión logística; ^§^IC 95% = Intervalo de confianza; ^||^p-valor = prueba de Wald, al nivel del 5%; ^¶^Salario mínimo R$ 1.212,00, Brasil, 2022; **DM = Diabetes Mellitus

## Discusión

Los resultados del presente estudio apuntan a una baja completitud de las vacunas, si se considera la lista de vacunas recomendadas para personas con DM. La mayoría no tenía ninguna dosis de la vacuna contra la hepatitis B y solo el 21,3% tenía calendario de vacunación completo (tres dosis), así como en otros estudios que evaluaron la vacunación en personas con DM^(10,12-14)^. Esta situación es preocupante, ya que estas personas presentan elevados índices de hepatitis B^(4)^ y los brotes de esta enfermedad se han asociado con procedimientos de monitoreo de glucemia entre personas con DM^(16)^.

En cuanto a la vacunación contra la influenza, la mayoría presentó una dosis única, la cual se administra anualmente. Este hallazgo puede estar relacionado con las campañas que se realizan cada año, con los llamamientos en los medios de comunicación, así como con la inclusión de la DM en el grupo de enfermedades crónicas prioritarias para recibir la vacuna en el 2013. Pese a ello, la meta de cobertura de vacunación del 90% en el país no se alcanzó en el 2022^(17)^.

En el presente estudio, se identificó que los adultos jóvenes (20 a 59 años) presentaron menor probabilidad de vacunarse en comparación con las personas ancianas, hallazgo que corrobora otros estudios^(11-12,14)^, que mostraron una mayor frecuencia de vacunación contra la influenza en personas mayores de 65 y 60 años, respectivamente. Se trata de una estrategia de prevención fundamental para este rango de edad, ya que las ocurrencias más frecuentes de SARS por influenza en Brasil, en el período del 2021, ocurrieron entre personas de 60 años o más, lo que representa el 52,1% (2.252) de los casos^(17)^.

Un estudio de cohorte retrospectivo de Estados Unidos constató que las personas con infección por influenza tenían tasas notablemente aumentadas de neumonía, cardiopatía isquémica y sepsis en la periinfluenza en comparación con los períodos iniciales. Para las personas con DM2, estos resultados sugieren que, para ellas, la influenza aumenta el riesgo de desenlaces clínicos más graves y de utilización de los cuidados de salud en comparación con la población base^(18)^.

La mayoría de las personas con DM que participó en el presente estudio tampoco contaba con calendario de vacunación antineumocócica, hallazgo similar al encontrado en una investigación que evaluó esta vacuna en personas con DM atendidas en una unidad de referencia del Distrito Federal, así como el nivel de conocimiento de los profesionales sobre el tema y el impacto de las orientaciones en la adhesión a la vacunación. Se constató que solo el 12,1% de las personas con DM y neumonía reportó la vacunación antineumocócica, el 69,7% la negó y el 18,2% desconocía su existencia^(19)^.

En cuanto a las causas y debilidades que afectan la cobertura vacunal antineumocócica, se observa que existe desinformación entre los profesionales prescriptores de la APS sobre las recomendaciones del Ministerio de Salud (MS). En este contexto, se requieren estrategias como mejorar la capacitación de los profesionales de la APS sobre las recomendaciones del MS para la vacunación de adultos, optimizar el flujo de solicitud y dispensación de las vacunas, simplificar los procesos de información, promover la participación multiprofesional y concienciar tanto a los profesionales como a las personas usuarias de la importancia de la vacunación^(20)^.

Cabe señalar que se trata de una vacuna disponible en los CRIE mediante prescripción médica. Por lo tanto, dos acciones podrían incrementar el acceso y la adhesión de las personas con DM a la vacuna antineumocócica: primero, informar a los profesionales prescriptores sobre la importancia de solicitar a los CRIE esta vacuna para las personas con DM; y segundo, ampliar la lista de profesionales que pueden solicitar/prescribir la vacuna para personas con DM.

Un ensayo clínico aleatorizado evaluó las tasas de vacunación de personas con DM que recibieron orientación sobre la actualización de su esquema vacunal contra influenza, hepatitis B, neumonía y tétanos. No se observó un aumento significativo, lo que demuestra que estas personas no recibieron una orientación adecuada por parte de los profesionales que las atendieron. Es fundamental promover acciones educativas entre los profesionales de la salud, concienciándolos de la relevancia de la orientación sobre la vacunación para personas con DM, para que comiencen a realizarla durante las consultas de rutina^(21)^.

En lo que respecta a la vacunación contra COVID-19, la mayoría presentó el calendario completo. Las personas jóvenes, con menos tiempo viviendo con DM y DM1, presentaron menos probabilidades de vacunarse en comparación con las persona mayores, quienes habían vivido más tiempo con la enfermedad y las que tenían DM2. Las personas mayores tienen más información sobre salud, dado que, además de asistir a más servicios de salud que las jóvenes, las campañas de vacunación contra la COVID-19 han enfatizado la relevancia de una protección específica para los grupos de mayor riesgo debido al mal pronóstico de la infección en las personas ancianas.

Las personas con DM tienen una mayor vulnerabilidad a enfermedades graves por COVID-19 en comparación con las que no tienen DM. En estas personas, las posibilidades de hospitalización relacionada con la COVID-19 y la gravedad de la enfermedad son de tres a cuatro veces mayores en comparación con las que no tienen DM^(22)^. Por lo tanto, la prevención primaria, mediante la vacunación oportuna, sigue siendo la base para mitigar los riesgos asociados a la COVID-19 en personas con DM^(23)^.

Hubo poquísima vacunación contra la difteria y el tétanos. Se sabe que las personas con DM2 son propensas a la infección por tétanos, especialmente cuando se les diagnostica una lesión de “pie diabético”, porque la vasculatura capilar reducida en las extremidades inferiores se convierte en una infección polimicrobiana complicada con bacterias anaeróbicas^(8)^.

Respecto a la vacuna contra la varicela, a pesar de ser sumamente recomendada por las SBD y SBIM^(6)^, también presentó una cobertura insignificante. Para las personas con DM, este inmunobiológico solo está disponible en los CRIE, por lo que se debe sensibilizar a los profesionales prescriptores de la importancia de esta vacuna para dicho público. Alrededor del 98% de los adultos brasileños, al llegar a los 60 años, tiene antecedentes de varicela y pueden desarrollar herpes zóster^(6)^. Como resultado, estas personas presentan un riesgo sustancialmente mayor de desarrollar herpes zóster, además de sus complicaciones como el dolor causado por la fase aguda y la neuralgia posherpética^(24)^.

En cuanto a la percepción de las personas con DM sobre la vacunación, la mayoría nunca había oído hablar del PNI, que es reconocido como una de las mayores políticas públicas de acceso a la vacunación en el ámbito mundial. En Brasil, este programa se difunde como una importante estrategia de salud, sin embargo, podría estar más propagado entre la población brasileña adulta y anciana, especialmente por parte de los profesionales que actúan en la APS.

En esta investigación uno de los motivos más representativos para la incompletitud del esquema vacunal fue no haber sido informado por el profesional de la salud sobre la importancia de vacunarse. Se sabe que la recomendación del profesional de la salud es una estrategia fundamental en la decisión de aceptar la vacunación^(21,25)^. Se debe proporcionar información clara y accesible sobre el funcionamiento de los servicios de vacunación, incluidos sus respectivos calendarios y campañas^(9)^.

Los profesionales de la salud que más asesoraron a las personas con DM sobre la importancia de vacunarse fueron los ACS y los enfermeros. Cabe resaltar que estos profesionales tienen un contacto más frecuente con personas con DM y deben aprovechar el momento oportuno para orientarles acerca de los beneficios de mantener el calendario de vacunación al día.

Se enfatiza la importancia de una comunicación efectiva, que no solo transmita información sobre la relevancia de la vacunación como medida preventiva, sino que también promueva la seguridad de las personas con relación a las vacunas. Además, es fundamental fortalecer la credibilidad del PNI entre la población^(26)^, para evidenciar los beneficios de la vacunación, así como para aclarar dudas que impactan en la decisión de vacunarse.

Entre los motivos para la incompletitud del esquema vacunal, se mencionan la falta de comprensión sobre la importancia de la vacunación y la falta de información proporcionada por los profesionales. Se sabe que dichos agentes pueden facilitar la adhesión a la vacunación, fomentando el proceso y tranquilizando a las personas en cuanto a la seguridad y a los efectos adversos^(9)^. Una investigación^(26)^ señaló que el miedo a eventos adversos compromete el acceso a la vacunación, además de otros factores socioeconómicos y geográficos. Comprender estos factores es esencial para planificar estrategias de vacunación adaptadas a las disparidades existentes y a la necesidad de políticas más equitativas en este contexto.

Por lo tanto, es fundamental intensificar las campañas, brindar aclaraciones y oportunizar la práctica de la vacunación entre las personas usuarias, especialmente en la población adulta joven, quienes se muestran más resistentes a buscar servicios de salud. Desde esta perspectiva, destaca el papel del enfermero como educador en salud, estableciendo canales de comunicación para difundir información sobre la vacunación y potenciar la adhesión^(27)^.

En lo que se refiere a todas las vacunas, la mayoría de la población con DM fue vacunada en los establecimientos de salud del SUS, y las USF fueron los lugares más representativos. Esto consolida al PNI como un programa de acceso universal y fundamental a la Salud Pública, asegurando que los inmunobiológicos esenciales estén disponibles para toda la población, independientemente de su condición socioeconómica o geográfica. Además, se relaciona el hecho de la ampliación de la cobertura poblacional de la APS.

Entre los obstáculos para la vacunación, los más reportados fueron las dificultades para desplazarse debido a limitaciones físicas o a la falta de transporte, así como la escasez de insumos o vacunas al momento de la inmunización. Se observa que esta población está compuesta predominantemente por personas ancianas, muchas de las cuales se enfrentan a algún grado de limitación física debido al envejecimiento y tienen bajos ingresos, lo que dificulta el acceso a los servicios de salud.

Ante ello, los establecimientos de salud, especialmente en la APS, deben implementar estrategias para llegar a las personas con dificultades de acceso, así como articular los servicios de atención domiciliaria para garantizar la oferta de vacunación. Además, un elemento identificado que dificultó el acceso a la vacunación fue la falta de insumos/vacunas, por lo que es fundamental planificar el número de dosis necesarias. Aspectos relacionados con la organización y la planificación de campañas de vacunación pueden influir en la adhesión de las personas usuarias^(9)^.

Las limitaciones del estudio se refieren a la realidad local en la que se realizó la investigación, lo que dificulta generalizar los resultados a otras regiones. Por lo tanto, se recomienda realizar más investigaciones para comprender otros escenarios, así como los obstáculos relacionados con la práctica de la vacunación en la población con DM.

Respecto a los avances en el conocimiento científico, se trata del primer estudio nacional que analizó la situación vacunal de las personas con DM para las vacunas recomendadas por el PNI^(5-6)^, incluso contra la COVID-19, además de exponer sus percepciones sobre la vacunación y las dificultades que enfrenta la APS.

Así, el presente estudio permitió reflexionar sobre la importancia de la vacunación entre estas personas, considerando el estímulo a los profesionales de la salud, especialmente a los enfermeros, para orientar sobre la relevancia y crear momentos oportunos para la vacunación, como, por ejemplo, visitas para realizar procedimientos como la glucemia capilar. Además, se espera que este estudio dirija intervenciones que aborden las causas de la baja completitud de las vacunas, como la falta de orientación profesional, incluyendo la adopción y el fortalecimiento de prácticas como el llenado de la libreta de vacunación para las personas con DM y el uso del eSUS-APS para el registro de las dosis administradas.

## Conclusión

Se constató que las personas con DM 1 y 2 presentaron baja completitud de las vacunas en el contexto de la APS, según el listado de vacunas recomendadas por el PNI para este grupo poblacional estudiado. Cabe señalar que son datos preocupantes, teniendo en cuenta que se trata de personas en una situación de mayor vulnerabilidad a las infecciones inmunoprevenibles y a la mortalidad, con un empeoramiento de su calidad de vida si desarrollan una enfermedad que podría prevenirse.

Se recomienda aunar esfuerzos para comprender los desafíos del acceso a la vacunación y mejorar la situación vacunal de las personas con DM por medio de la educación permanente de los profesionales de la salud y una mayor concienciación pública. Se resalta el papel del equipo de enfermería durante todo el proceso de operativización de las acciones de vacunación en el país, lo que puede ser decisivo para mejorar la situación vacunal de las personas con DM.

# Translation: Situação vacinal de pessoas com diabetes *mellitus* atendidas na Atenção Primária à Saúde: estudo transversal*

**DOI:** 10.1590/1518-8345.7065.4453

**Authors:** Francisco João de Carvalho, Luisa Helena de Oliveira Lima, Mônica Antar Gamba, Rosilane Lima de Brito, Lucilane Maria Sales da Silva, Ana Roberta Vilarouca da Silva

**Affiliations:** 1 Universidade Estadual do Ceará, Fortaleza, CE, Brazil.; 2 Bolsista da Fundação Cearense de Apoio ao Desenvolvimento Científico e Tecnológico (FUNCAP), Brazil.; 3 Universidade Federal do Piauí, Teresina, PI, Brazil.; 4 Universidade Federal de São Paulo, São Paulo, SP, Brazil.

**Keywords:** Cobertura Vacinal, Diabetes Mellitus Tipo 2, Diabetes Mellitus Tipo 1, Atenção Primária à Saúde, Enfermagem, Vacinas

## Abstract

analisar a situação vacinal de pessoas com diabetes *mellitus* tipos 1 e 2 na Atenção Primária à Saúde.

estudo transversal, analítico, realizado em 25 equipes de Saúde da Família com 274 pessoas com diabetes. Foram avaliadas as variáveis sociodemográficas e clínicas e esquema vacinal completo para cada vacina recomendada pelo Programa Nacional de Imunizações, por meio de análise bivariada e regressão logística.

das pessoas com diabetes *mellitus*, 69,1% não tinham esquema vacinal completo contra hepatite B; 64,6%, contra difteria e tétano; 74,3%, contra febre amarela; 87,9%, contra pneumococo; 87,9%, contra varicela; 24,5%, contra influenza; e 0,7%, contra a covid-19. Entre os motivos referidos para o esquema incompleto, estão não conhecer a importância de vacinar e não ser informado por profissional de saúde. Houve associação estatística significativa entre o perfil sociodemográfico e clínico em relação ao esquema vacinal completo da vacina da influenza e a idade e renda; covid-19 e idade, tipo de diabetes e tempo convivendo com o diabetes.

pessoas com diabetes *mellitus* tipos 1 e 2 apresentaram baixa completude vacinal no contexto da Atenção Primária à Saúde, o que é preocupante devido à situação de maior vulnerabilidade a infecções imunopreveníveis e mortalidade nesse grupo.

## Destaques

**(1)** Pessoas com diabetes apresentam baixa completude vacinal.

**(2)** É fundamental oportunizar a vacinação nos serviços de saúde às pessoas com diabetes.

**(3)** A educação dos profissionais prescritores de vacinas é necessária.

**(4)** Jovens apresentam menos chance de se vacinar do que idosos.

**(5)** É imprescindível conhecer os fatores predisponentes à situação vacinal.

## Introdução

Estima-se que 537 milhões de adultos com idades entre 20 e 79 anos em todo o mundo têm diabetes *mellitus* (DM). Em 2030, a estimativa é de 643 milhões; e em 2045, 783 milhões. No cenário brasileiro, o país está entre os dez principais territórios em número de adultos (20 a 79 anos) com DM em 2021, ocupando a 6ª posição^(1)^.

Essa condição hiperglicêmica persistente interfere significativamente na efetividade do sistema imunológico dessas pessoas, pois há comprometimento dos mecanismos de defesa, como supressão da produção de citocinas, defeitos na fagocitose, disfunção de células imunológicas e incapacidade de deter microorganismos^(2-3)^. Isso as torna mais suscetíveis a determinadas doenças infecciosas^(3)^ que poderiam ser prevenidas com a vacinação, impactando a redução de morbidade e hospitalizações^(4)^.

A vacinação de pessoas com DM é estratégia fundamental de proteção da saúde e de promoção da qualidade de vida. Ressalta-se que essas pessoas devem ter atenção especializada, uma vez que têm chances aumentadas em: 2,5 vezes para desenvolver síndrome respiratória aguda grave (SRAG) por influenza; 1,4 vez para pneumonia adquirida na comunidade; 4,6 vezes para doença pneumocócica invasiva; 2 vezes para complicações hepáticas, quando também têm hepatite B crônica; e 3 vezes para herpes-zóster^(5)^.

Em consonância com as Sociedade Brasileira de Diabetes (SBD) e a Sociedade Brasileira de Imunizações (SBIm), e considerando as recomendações do Centro de Referência de Imunobiológicos Especiais (CRIE), as seguintes vacinas são altamente recomendadas para pessoas com DM: influenza, pneumocócica conjugada 13-valente (VPC13), pneumocócica polissacarídica 23-valente (VPP23), hepatite B, *Haemophilus influenzae*do tipo b (Hib), varicela e herpes-zóster, observando que a maioria está disponível no Programa Nacional de Imunizações (PNI)^(6)^.

Entre os imunobiológicos disponíveis na Atenção Primária à Saúde (APS) e nos CRIE estão as vacinas contra influenza, hepatite B, febre amarela, difteria e tétano adulto (dT), tríplice viral e covid-19. As vacinas pneumocócicas VPP23, conjugada 13-valente VPC13 e varicela, apesar de recomendadas, não estão disponíveis na APS para pessoas com DM; apenas nos CRIE, mediante prescrição médica. Já a vacina herpes-zóster, somente na rede privada.

A vacinação é pilar essencial da APS e representa um direito humano inquestionável. Nas unidades da rede de APS, é conduzida a verificação da caderneta e da situação vacinal, além da orientação e administração para iniciar ou completar o esquema vacinal, de acordo com os calendários específicos^(7)^. Ademais, o registro detalhado das vacinas recebidas por pessoas com DM, juntamente com a avaliação das suas necessidades, constituem elementos essenciais de uma avaliação clínica holística, realizada no início do acompanhamento e durante as consultas anuais^(7)^.

Os determinantes sociais influenciam a decisão de se vacinar. Entre os fatores socioeconômicos e clínicos associados à condição de estar vacinado, menores escolaridade e renda repercutem negativamente na vacinação, enquanto maior idade está associada a maiores taxas de vacinação^(8)^. Além desses, fatores logísticos e relacionados a profissionais de saúde podem ser barreiras à vacinação^(9)^.

No entanto, apesar das recomendações, estudos nacionais^(10-11)^ e internacionais^(12-14)^ que avaliaram a vacinação em pessoas com DM não contemplaram todas as vacinas recomendadas a tal grupo. Além de preencher essa lacuna, a presente pesquisa ainda aborda a percepção de pessoas com DM sobre a vacinação, bem como as respectivas dificuldades enfrentadas para se vacinarem. Diante disso, o planejamento e a implementação de estratégias para alcançar a população com DM são imprescindíveis e urgentes. Assim, objetivou-se analisar a situação vacinal de pessoas com diabetes *mellitus*tipos 1 e 2 na Atenção Primária à Saúde.

## Método

### Tipo de estudo

Estudo transversal, analítico, baseado nas diretrizes do *Strengthening the Reporting of Observational Studies in Epidemiology*(STROBE)^(15)^.

### Local

A pesquisa foi desenvolvida em um município do Piauí, Brasil, localizado no centro-sul do estado, com população estimada de 83 mil habitantes. Tem cobertura de APS de 100%, contando com 36 equipes de Saúde da Família (eSF), sendo 25 localizadas na zona urbana e 11 na zona rural. Tais equipes compõem as Unidades de Saúde da Família (USF), cujos estabelecimentos contemplam pelo menos uma eSF.

### Período

A coleta de dados ocorreu no período de janeiro a julho de 2022.

### População e amostra

A população foi composta por usuários cadastrados nas 25 eSF situadas na zona urbana do município estudado. O sistema de informação utilizado no cadastramento de pessoas com DM é o e-SUS APS. Utilizou-se o cálculo de amostras para população finita, estratificado por proporção, somando 2.564 pessoas com DM tipo 1 (DM1) e DM tipo 2 (DM2), com um erro amostral de 5% e nível de confiança de 95%. Para fim de prevalência, foi usado 0,5, objetivando obter a amostra máxima, considerando o nível de confiança de 95% e de significância de 5%. Uma vantagem que a amostragem estratificada proporcional permitiu diz respeito à distribuição proporcional das pessoas com DM cadastradas segundo o tamanho da população de cada eSF. Assim, a amostra final foi constituída por 274 pessoas com DM.

### Critérios de seleção

Foram incluídas pessoas com idade igual ou superior a 18 anos, com diagnóstico de DM1 ou DM2, cadastradas na USF de abrangência há pelo menos um ano. Foram excluídas aquelas que apresentaram algum déficit cognitivo que impossibilitasse resposta direta às perguntas do instrumento de coleta de dados, o que foi avaliado mediante aplicação do Miniexame do Estado Mental.

### Coleta de dados

Esta etapa foi conduzida por meio de aplicação do formulário de coleta de dados durante consultas de enfermagem às pessoas com DM que concordaram em participar da pesquisa, mediante a assinatura do Termo de Consentimento Livre e Esclarecido (TCLE). Para as pessoas que não frequentavam a USF, foram realizadas visitas domiciliares, acompanhadas por agentes comunitários de saúde (ACS). O processo cumpriu os seguintes passos: 1) Realização de reuniões com os ACS e enfermeiros, incluindo o sorteio dos participantes com base nas informações disponíveis sobre pessoas com DM em cada eSF; 2) Solicitação do número de telefone da pessoa sorteada para compor a amostra; 3) Agendamento da visita à residência por meio de contato telefônico prévio, no qual foram explicados o TCLE e o termo de entrada em domicílio; 4) Para aquelas pessoas que concordaram em participar, foi solicitado um ambiente apropriado no domicílio para a aplicação do instrumento de coleta de dados; 5) Foi utilizado o termo de entrada no domicílio para acessar a residência. Devido à pandemia da COVID-19, foram implementadas medidas protetivas para garantir a segurança tanto dos pesquisadores quanto dos entrevistados.

### Instrumento de coleta e variáveis do estudo

Elaborou-se instrumento de coleta de dados intitulado “Situação vacinal de pessoas com diabetes na APS” pelo pesquisador, tendo como base as recomendações da SBD e da SBIm e revisão de literatura sobre a temática. Contém 30 itens, com questões de múltipla escolha e abertas, divididos em três dimensões: caracterização sociodemográfica e clínica (16 itens), que abrange variáveis como sexo, escolaridade, renda, cor, religião, ocupação, tipo de DM, tempo convivendo com o DM em anos, tratamento medicamentoso e não medicamentoso, comorbidades, se tomava sempre a medicação, se tinha hábitos tabágicos e etilistas e se praticava exercício físico regularmente (mais de três vezes na semana).

A dimensão acerca da situação vacinal (três itens) avalia se a pessoa tem cartão de vacina, se ele foi visualizado e o esquema vacinal completo para hepatite b, influenza, dT, tríplice viral, febre amarela, contra a covid-19, varicela e pneumocócicas conjugadas e o local em que recebeu a vacina (USF, atenção secundária, domicílio ou CRIE).

A última dimensão diz respeito à percepção das pessoas com DM sobre vacinação (11 itens), que contemplou: conhecimento sobre o PNI; se consideram as campanhas midiáticas realizadas pelo PNI suficientes para motivar a vacinação; razão pela qual o esquema vacinal estava incompleto (medo de apresentar reações adversas, desconhecimento sobre importância de vacinar, experiência prévia de reações vacinais em decorrência de alguma vacina, não julgar necessário, ausência de informação pelo profissional de saúde, outros motivos – especificar). Os participantes poderiam responder a mais de um item. Além disso, questionava-se sobre a orientação do profissional de saúde acerca da importância de manter o esquema vacinal atualizado; seguimento das orientações sobre vacinação que os profissionais de saúde forneciam; retorno à USF para completar o esquema das vacinas de duas ou mais doses; efeito adverso pós-vacina; problema de saúde que impedisse de tomar vacina; orientação médica/enfermagem para não tomar mais vacina após esse problema de saúde; dificuldades para vacinar (USF muito longe, falta de tempo para ir, horário de funcionamento da unidade de saúde inadequada, mandaram voltar outro dia, não acho necessário, ao chegar não tinha insumos, não era do grupo prioritário naquele dia); e se alguma vez foi vacinar e não conseguiu.

Quanto às variáveis, a dependente foi a situação vacinal completa de cada vacina, e as independentes foram sexo, idade (em anos), escolaridade, situação conjugal, tempo de diabetes (anos). Consideraram-se vacinadas contra a influenza pessoas que apresentaram registro de uma dose da vacina de até um ano anterior à coleta; contra a hepatite B, registro de três doses; da tríplice viral, duas doses (20 a 29 anos) e uma dose (30 a 59 anos); febre amarela, registro de uma dose única; dT, três doses tomadas em até dez anos anteriores à coleta; pneumocócica conjugada (VPC13); varicela, duas doses; COVID-19, duas doses e um reforço.

### Tratamento e análise de dados

Os dados foram tabulados e organizados em planilha eletrônica do *Microsoft Excel*® 2016 e analisados utilizando o *software Statistical Package for the Social Sciences* (SPSS) versão 26. Para as análises descritivas exploratórias, foram aplicadas a frequência absoluta e relativa para variáveis qualitativas e as medidas de posição e dispersão para variáveis quantitativas. O intervalo de confiança foi aplicado para a frequência relativa e média das variáveis analisadas. Para verificar os fatores associados à condição de estar vacinado, foi realizada análise bivariada por meio do teste exato de Fisher (X^2^). Para as variáveis que apresentaram significância ao nível de 5%, foi aplicada a regressão logística bivariada para calcular a razão de chance ou *odds ratio* (OR). Foi adotado nível de significância de 5% (0,05), considerando valores de p < 0,05 como estatisticamente significantes para associação.

### Aspectos éticos

A pesquisa foi aprovada pelo Comitê de Ética em Pesquisa da instituição proponente sob número 5.036.594, em 14 de outubro de 2021, sendo conduzida, assim, em consonância com a Resolução 466/12 do Conselho Nacional de Saúde.

## Resultados

Das 274 pessoas com DM que compuseram a amostra, a maioria tinha 60 anos ou mais (169; 62,6%), era do sexo feminino (192; 70,1%), com renda menor ou igual a um salário-mínimo (150; 63,6%) e tinha ensino fundamental completo (139; 51,5%). Sobre as características clínicas, o tipo de DM mais relatado foi DM2 (260; 95, 2%), convivendo há mais de dez anos com a condição crônica (109; 41,3%), em uso de antidiabéticos orais como tratamento medicamentoso (231; 85,9%), conforme apresentado na Tabela 1.

A Tabela 2 evidencia que a maioria possuía cartão de vacina (262; 95,6%), sem nenhuma dose de hepatite B (188; 69,1%) e com esquema para a vacina contra a influenza (204, 75%). A maioria não tinha esquema vacinal contra: difteria e tétano (175; 64,6%), febre amarela (202; 74,3%), tríplice viral (223; 82,3%), pneumocócica (240; 87,9%) e varicela (239; 87,9%). A maior parte apresentou esquema completo para a COVID-19 (271; 68,5%). Quanto ao local de recebimento da vacina, as USF foram os mais citados (168; 62%).

**Tabela 1 -:** Caracterização do perfil sociodemográfico e clínico das pessoas com diabetes *mellitus* cadastradas nas eSF* (n = 274). Picos, PI, Brasil, 2022

Perfil sociodemográfico	n(%)	IC-95%^†^
Sexo		
Feminino	192(70,1)	(64,5-75,3)
Masculino	82(29,9)	(24,7-35,5)
Faixa etária		
20-59 anos	101(37,4)	(31,8-43,3)
≥ 60 anos	169(62,6)	(56,7-68,2)
Situação conjugal		
Solteiro(a)/Viúva(a)	126(46,7)	(40,8-52,6)
Casado(a)/União estável(a)	144(53,3)	(47,4-59,2)
Escolaridade		
Analfabeto(a)	58(21,5)	(16,9-26,7)
Ensino Fundamental	139(51,5)	(45,5-57,4)
Ensino Médio	56(20,7)	(16,2-25,9)
Ensino Superior	15(5,6)	(3,3-8,8)
Alfabetização	2(0,7)	(0,2-2,4)
Raça/cor da pele		
Branco	119(45,2)	(39,3-51,3)
Preto	57(21,7)	(17,0-26,9)
Pardo	86(32,7)	(27,2-38,5)
Outros	1(0,4)	(0,0-1,8)
Religião		
Católico(a)	215(82,4)	(77,4-86,6)
Evangélico(a)	35(13,4)	(9,7-17,9)
Outra	11(4,2)	(2,3-7,2)
Renda (salários-mínimos)^‡^		
≤ 1 SM	150(63,6)	(57,3-69,5)
> 1 SM	86(36,4)	(30,5-42,7)
Perfil Clínico		
Tipo de DM^§^		
Tipo 1	13(4,8)	(2,7-7,8)
Tipo 2	260(95,2)	(92,2-97,3)
Tempo convivendo com DM^§^		
< 5 anos	75(28,4)	(23,2-34,1)
5-10 anos	80(30,3)	(25,0-36,0)
> 10 anos	109(41,3)	(35,5-47,3)
Tipo de tratamento medicamentoso		
Antidiabéticos orais	231(85,9)	(81,3-89,6)
Insulina	24(8,9)	(6,0-12,8)
Ambos	14(5,2)	(3,0-8,4)
Tipo de tratamento não medicamentoso		
Exercícios físicos	31(11,4)	(8,0-15,5)
Educação alimentar	81(29,7)	(24,5-35,3)
Exercícios físicos e educação alimentar	54(19,8)	(15,4-24,8)
Nenhum	107(39,2)	(33,5-45,1)
Medicação		
Sempre	253(97,3)	(94,8-98,8)
Raramente	4(1,5)	(0,5-3,6)
Apenas com sintomas	3(1,2)	(0,3-3,0)
Comorbidades		
Não	49(17,9)	(13,7-22,7)
Sim	225(82,1)	(77,3-86,3)
Tabagismo		
Sim	28(10,2)	(7,1-14,2)
Não	222(81,0)	(76,1-85,3)
Já fui	24(8,8)	(5,8-12,5)
Etilismo		
Sim	28(10,2)	(7,1-14,2)
Não	200(73,0)	(67,5-78,0)
Já fui, mas não sou mais	46(16,8)	(12,7-21,6)
Exercício físico regularmente (mais que três vezes por semana)		
Sim	71(25,9)	(21,0-31,3)
Não	203(74,1)	(68,7-79,0)

*eSF = Equipe de Saúde da Família; ^†^IC-95% = Intervalo de confiança; ^‡^Salário-mínimo: R$1.212.00, Brasil, 2022; ^§^DM = Diabetes *mellitus*

**Tabela 2 -:** Situação vacinal das pessoas com diabetes *mellitus* cadastradas nas eSF* (n = 274). Picos, PI, Brasil, 2022

Situação vacinal	n (%)	IC-95%^†^
Possui cartão vacinal		
Sim	262(95,6)	(92,7-97,6)
Não	8(2,9)	(1,4-5,4)
Nunca possuiu	1(0,4)	(0,0-1,7)
Já possuiu, mas perdeu	3(1,1)	(0,3-2,9)
Cartão de vacina visualizado		
Sim	215(78,5)	(73,3-83,0)
Não	59(21,5)	(17,0-26,7)
Se não, o esquema vacinal foi:		
Autorreferido	57(98,3)	(92,2-99,8)
Não sabia	1(1,7)	(0,2-7,8)
Possui esquema vacinal para hepatite B		
Sim	58(21,3)	(16,8-26,5)
Não	188(69,1)	(63,4-74,4)
Não sei	26(9,6)	(6,5-13,5)
Possui esquema vacinal para influenza		
Sim	204(75,0)	(69,6-79,9)
Não	64(23,5)	(18,8-28,8)
Não sei	4(1,5)	(0,5-3,5)
Possui esquema vacinal para dT^‡^		
Sim	69(25,5)	(20,6-30,9)
Não	175(64,6)	(58,8-70,1)
Não sei	27(10,0)	(6,8-14,0)
Possui esquema vacinal para tríplice viral		
Sim	18(6,6)	(4,1-10,1)
Não	223(82,3)	(77,4-86,5)
Não sei	30(11,1)	(7,8-15,2)
Possui esquema vacinal para febre amarela		
Sim	42(15,4)	(11,5-20,1)
Não	202(74,3)	(68,8-79,2)
Não sei	28(10,3)	(7,1-14,3)
Possui esquema vacinal contra a COVID-19		
Sim	271(99,3)	(97,7-99,8)
Não	2(0,7)	(0,2-2,3)
Não sei	0(0,0)	
Teve reações adversas a essa vacina		
Sim	115(43,1)	(37,2-49,1)
Não	152(56,9)	(50,9-62,8)
Possui esquema vacinal para varicela		
Sim	4(1,5)	(0,5-3,5)
Não	239(87,9)	(83,6-91,3)
Não sei	29(10,7)	(7,4-14,7)
Possui esquema vacinal para pneumocócicas conjugadas (VPC10^§^, VPC13^||^)		
Sim	4(1,5)	(0,5-3,4)
Não	240(87,9)	(83,7-91,4)
Não sei	29(10,6)	(7,4-14,7)
Local em que recebeu a vacina		
USF^¶^	168(62,0)	(56,1-67,6)
Domicílio	2(0,7)	(0,2-2,3)
Atenção Secundária	101(37,3)	(31,7-43,1)
CRIE**	0(0,0)	

*eSF = Equipe de Saúde da Família; ^†^IC-95% = Intervalo de confiança; ^‡^dT = Vacina dupla adulto; ^§^VPC10 = Vacina pneumocócica conjugada 10-valente; ^||^VPC13 = Vacina pneumocócica conjugada 13-valente; ^¶^USF = Unidade de Saúde da Família; **CRIE = Centro de Referência de Imunobiológicos Especiais

A maioria dos entrevistados (165; 60,2%) afirmou nunca ter ouvido falar do PNI. Além disso, quase metade (129; 47,1%) considerou que as campanhas realizadas pelo programa por meio da mídia não são suficientes para motivar o público a se vacinar. Os principais motivos mencionados para a incompletude do esquema vacinal foram não conhecer a importância da vacinação (68; 25,9%) e não ser informado pelo profissional de saúde (98; 37,3%), como é apresentado na Tabela 3.

**Tabela 3 -:** Percepção das pessoas com diabetes *mellitus* cadastradas nas eSF* sobre vacinação (n = 274). Picos, PI, Brasil, 2022

Percepção sobre vacinação	n (%)	IC-95%^†^
Conhecimento do PNI^‡^		
Sim	109(39,8)	(34,1-45,7)
Não	165(60,2)	(54,3-65,9)
Considera as campanhas realizadas pelo PNI^‡^, por meio da mídia, suficientes como motivação para vacinar-se		
Sim	108(40,4)	(34,7-46,4)
Não	129(48,3)	(42,4-54,3)
Parcialmente	30(11,2)	(7,9-15,4)
Motivo do esquema vacinal incompleto		
Medo das reações adversas	21(8,0)	(5,2-11,7)
Não conhece a importância de vacinar	68(25,9)	(20,8-31,4)
Descuido	64(24,3)	(19,4-29,8)
Acho que não preciso	7(2,7)	(1,2-5,2)
Não foi informado(a) pelo profissional de saúde	98(37,3)	(31,6-43,2)
Outros	5(1,9)	(0,7-4,1)
Recebeu orientação acerca da importância de manter o esquema vacinal atualizado		
Sim	208(76,2)	(70,9-80,9)
Não	65(23,8)	(19,1-29,1)
Segue as orientações sobre vacinação fornecidas pelos profissionais de saúde		
Sim	178(68,5)	(62,6-73,9)
Não	46(17,7)	(13,4-22,7)
Parcialmente	36(13,8)	(10,1-18,4)
Efeito adverso após vacinação		
Sim	138(50,7)	(44,8-56,6)
Não	134(49,3)	(43,4-55,2)
Para as vacinas que precisam de retorno para completar o esquema, volta à USF^§^		
Sim	220(83,0)	(78,2-87,2)
Não	45(17,0)	(12,8-21,8)
Tem problema de saúde que impeça de tomar vacina		
Sim	3(1,1)	(0,3-2,9)
Não	271(98,9)	(97,1-99,7)
Orientação (médica/enfermagem) para não tomar mais vacina após esse problema de saúde		
Sim	1(0,4)	(0,0-1,7)
Não	272(99,6)	(98,3-100,0)
Dificuldade para vacinar		
Sim	67(24,5)	(19,6-29,8)
Não	207(75,5)	(70,2-80,4)
Alguma vez foi vacinar e não conseguiu		
Sim	30(11,0)	(7,7-15,2)
Não	242(89,0)	(84,8-92,3)

*eSF = Equipes de Saúde da Família; ^†^IC-95% = Intervalo de confiança; ^‡^PNI = Programa Nacional de Imunizações; ^§^USF = Unidade de Saúde da Família

Nesta pesquisa, as principais dificuldades relatadas pelas pessoas com DM para a vacinação incluem dificuldade de deslocamento (35; 52,2%) e escassez de insumos/vacinas (22; 32,8%), conforme a Figura 1.

**Figura 1 -:** Principais dificuldades para vacinação das pessoas com diabetes *mellitus* atendidas na eSF (n = 65). Picos, PI, Brasil, 2022

Na Tabela 4, foi analisada a razão de chance entre as variáveis que apresentaram significância estatística (p<0,005) na análise bivariada. Assim, observou-se que a vacinação contra a influenza esteve associada à idade (OR=0,395; IC206-0,755), às religiões católica (OR=6,275; IC1,201-32,781) e evangélica (OR= 5,295; IC0,878-31,950), à renda familiar (OR=0,321; IC0,144-0,718) e ao etilismo (OR=0,394; IC,0,148-1,050).

No caso da vacina tríplice viral, o tipo de DM não influenciou as chances da pessoa com DM se vacinar (OR=8,491); já aquelas em uso de antidiabéticos orais têm menos chances de se vacinar em relação aos que fazem uso de ambos os tratamentos (insulina e antidiabéticos orais) (OR=0,313; IC0,028-3,515). Quanto à febre amarela, pessoas com DM1 têm mais chances de se vacinar do que aquelas com DM2 (OR=4,286; IC1,292-14,218).

Em relação à vacinação contra a covid-19, pessoas mais jovens (20 a 59 anos) têm menos chances de apresentarem esquema vacinal do que pessoas idosas (60 anos ou mais) (OR=0,342; IC0,153-0,761); pessoas com DM1 foram mais propensas a se vacinar do que aquelas com DM2 (0R=0,142; IC0,029-0,753); e quanto ao tempo de convívio com DM, pessoas com menos anos têm menos chances de completar o esquema vacinal (OR=0,132; IC0,039-0,445) em relação a quem convive há mais tempo com a condição (OR=0,276; IC0,078-0,968).

**Tabela 4 -:** Análise de regressão logística da associação entre esquema vacinal de influenza, dT*, tríplice viral, febre amarela e covid-19 e perfil sociodemográfico e clínico das pessoas com diabetes *mellitus* cadastradas nas eSF^†^ (n = 274). Picos, PI, Brasil, 2022

**Vacina/Variável**	**OR** ^‡^	**(IC-95%)** ^§^	**p-valor** ^||^
**Influenza**			
**Faixa etária**			
20-59 anos	0,395	(0,206-0,755)	0,005
≥ 60 anos			
**Religião**			
Católico(a)	6,275	(1,201-32,781)	0,029
Evangélico(a)	5,295	(0,878-31,950)	0,069
Outra			
**Renda familiar (salários-mínimos)** ^¶^			
≤1 SM	0,321	(0,144-0,718)	0,006
>1 SM			
**Etilismo**			
Sim	0,394	(0,148-1,050)	0,062
Não	1,508	(0,728-3,124)	0,268
Já fui, mas não sou mais			
dT*			
Tempo convivendo com o DM**			
< 5 anos	3,429	(0,779-15,086)	0,103
5-10 anos	0,429	(0,123-1,491)	0,183
> 10 anos			
**Tríplice viral**			
**Escolaridade**			
Analfabeto(a)	0,055	(0,003-1,102)	0,058
Ensino Fundamental	0,030	(0,002-0,576)	0,020
Ensino Médio	0,143	(0,008-2,552)	0,186
Ensino Superior	0,250	(0,012-5,262)	0,373
Tipo de DM**			
Tipo 1	8,491	(0,766-94,064)	0,081
Tipo 2			
**Tratamento medicamentoso**			
Antidiabéticos orais	0,187	(0,039-0,899)	0,036
Insulinoterapia	0,313	(0,028-3,515)	0,346
Ambos			
**Exercício fisíco regularmente (mais de três vezes/semana)**			
Sim	0,108	(0,013-0,894)	0,108
Não			
**Febre amarela**			
Tipo de DM**			
Tipo 1	4,286	(1,292-14,218)	0,017
Tipo 2			
**COVID-19**			
**Faixa etária**			
20-59 anos	0,342	(0,153-0,761)	0,09
≥ 60 anos			
Tipo de DM**			
Tipo 1	0,142	(0,029-0,753)	0,021
Tipo 2			
Tempo convivendo com o DM**			
< 5 anos	0,132	(0,039-0,445)	0,001
5-10 anos	0,276	(0,078-0,968)	0,044
> 10 anos			

*dT = Vacina dupla adulto; ^†^eSF = Equipes de Saúde da Família; ^‡^OR = Regressão logística; ^§^IC-95% = Intervalo de confiança; ^||^p-valor = Teste de Wald, ao nível de 5%; ^¶^Salário-mínimo: R$1.212.00, Brasil, 2022; **DM = Diabetes *mellitus*

## Discussão

Os resultados do presente estudo apontam a baixa completude vacinal ao se considerar o rol de vacinas recomendadas para pessoas com DM. A maioria não tinha nenhuma dose da vacina hepatite B, e apenas 21,3% apresentaram esquema vacinal completo (três doses), assim como em outros estudos que avaliaram vacinação de pessoas com DM^(10,12-14)^. Tal situação revela-se preocupante, uma vez que essas pessoas apresentam taxas elevadas de hepatite B^(4)^, e surtos dessa doença têm sido associados a procedimentos de monitoramento da glicemia entre pessoas com DM^(16)^.

Quanto à vacinação contra a influenza, a maioria apresentou a dose única, administrada anualmente. Esse achado pode ter relação com campanhas que são realizadas todos os anos, aos apelos midiáticos, bem como a inclusão do DM no grupo de doenças crônicas prioritárias para o recebimento da vacina em 2013. Apesar disso, a meta de 90% de cobertura vacinal no país não foi alcançada no ano de 2022^(17)^.

No presente estudo, identificou-se que adultos jovens (20 a 59 anos) apresentaram menor chance de se vacinar que pessoas idosas, cujo achado corrobora outros estudos^(11-12,14)^, os quais apontaram maior frequência de vacinação contra influenza em pessoas com mais de 65 e 60 anos, respectivamente. Trata-se de uma estratégia de prevenção imprescindível para essa faixa etária, já que a mais frequente dentre as ocorrências de SRAG por influenza no Brasil, no período de 2021, foi entre pessoas com 60 anos ou mais, representando 52,1% (2.252) dos casos^(17)^.

Estudo de coorte retrospectiva dos Estados Unidos constatou que pessoas com infecção por influenza apresentaram taxas acentuadamente aumentadas de pneumonia, doença cardíaca isquêmica e sepse na peri-influenza em comparação com os períodos basais. Já em pessoas com DM2, esses resultados sugerem que a influenza aumenta o risco de desfechos clínicos mais graves e utilização de cuidados de saúde em comparação com a população base^(18)^.

A maioria das pessoas com DM participantes do presente estudo também não apresentava esquema para a vacina antipneumocócica, achado semelhante ao encontrado em investigação que avaliou tal vacina em pessoas com DM atendidas em unidade de referência no Distrito Federal, bem como o nível de conhecimento dos profissionais de saúde sobre o assunto e o impacto da orientação na adesão à vacinação. Constatou-se que apenas 12,1% das pessoas com DM e pneumonia informaram vacinação antipneumocócica, 69,7% negaram, e 18,2% não sabiam da sua existência^(19)^.

Em relação às causas e fragilidades que afetam a cobertura vacinal antipneumocócica, observa-se que há desinformação entre os profissionais prescritores na APS sobre as recomendações do Ministério da Saúde (MS). Nesse contexto, estratégias como aprimorar a capacitação dos profissionais da APS sobre as recomendações do MS para a vacinação de adultos, otimizar o fluxo de solicitação e dispensação das vacinas, simplificar os processos de informação, promover o envolvimento multiprofissional e conscientizar tanto os profissionais quanto as pessoas usuárias sobre a importância da vacinação se mostram necessárias^(20)^.

Ressalta-se que essa é uma vacina disponível nos CRIEs a partir da prescrição do médico. Dessa forma, duas ações poderiam ampliar o acesso e a adesão vacinal das pessoas com DM para a antipneumocócica: primeiro, informar aos profissionais prescritores sobre a importância de solicitarem essa vacina à pessoa com DM junto aos CRIEs; e segundo, ampliar o rol de profissionais que podem solicitar/prescrever a vacina para as pessoas com DM.

Um ensaio clínico randomizado avaliou as taxas de vacinação de pessoas com DM que receberam orientação para atualização do calendário vacinal contra influenza, hepatite B, pneumonia e tétano. Não foi observado aumento significativo, demonstrando que tais pessoas não receberam orientações adequadas dos profissionais que os atendiam. Torna-se essencial promover ações educativas junto aos profissionais de saúde, conscientizando-os a respeito da relevância da orientação sobre vacinação para pessoas com DM, de modo que passem a realizá-la nas consultas de rotina^(21)^.

Concernente à vacinação contra a covid-19, a maioria apresentou esquema completo. Pessoas jovens, com menos tempo de convívio com DM e DM1, apresentaram menos chances de se vacinar em comparação às pessoas idosas, àquelas que viviam há mais tempo com a condição e às que tinham DM2. Pessoas idosas têm mais informação sobre saúde, pois, além de frequentarem mais os serviços de saúde que as jovens, as campanhas de vacinação contra a covid-19 enfatizaram a relevância de proteção específica aos grupos de maior risco devido ao mau prognóstico da infecção em pessoas idosas.

Pessoas com DM têm maior vulnerabilidade a doenças graves por covid-19 do que aquelas sem DM. Em tais pessoas, as chances de hospitalização relacionada à covid-19 e maior gravidade da doença são de três a quatro vezes maiores em comparação àquelas sem DM^(22)^. Assim, a prevenção primária, por meio da vacinação em tempo oportuno, continua sendo a base para mitigar os riscos associados à covid-19 em pessoas com DM^(23)^.

Houve baixíssima vacinação contra difteria e tétano. Sabe-se que pessoas com DM2 estão propensas a infecção por tétano, especialmente quando diagnosticadas com lesão em “pé diabético”, porque a vasculatura capilar reduzida nas extremidades inferiores se transforma em uma infecção polimicrobiana complicada com bactérias anaeróbicas^(8)^.

Em relação à vacina contra varicela, apesar de altamente recomendada pela SBD e pela SBIm^(6)^, também apresentou cobertura ínfima. Para pessoas com DM, esse imunobiológico está disponível apenas nos CRIEs, devendo-se, portanto, sensibilizar os profissionais prescritores acerca da importância dessa vacina para o referido público. Cerca de 98% dos adultos brasileiros, ao chegar aos 60 anos, têm histórico de varicela, podendo desenvolver herpes-zóster^(6)^. Essas pessoas apresentam risco substancialmente aumentado de desenvolvimento de herpes-zóster, além de suas complicações como a dor causada pela fase aguda e a neuralgia pós-herpética^(24)^.

Com relação à percepção das pessoas com DM sobre vacinação, a maioria nunca tinha ouvido falar do PNI, o qual é reconhecido como uma das maiores políticas públicas de acesso a vacinação em nível mundial. No Brasil, tal programa é difundido como uma importante estratégia da saúde, contudo poderia ser mais propagado entre a população brasileira adulta e idosa, especialmente pelos profissionais atuantes na APS.

Nessa investigação, um dos motivos mais representativos para o esquema vacinal incompleto foi não ser informado pelo profissional de saúde sobre a importância de se vacinar. Sabe-se que a recomendação do profissional de saúde é uma estratégia fundamental na decisão de aceitar a vacinação^(21,25)^. Deve fornecer informações claras e acessíveis quanto ao funcionamento dos serviços de vacinação, incluindo os respectivos calendários vacinais e as campanhas^(9)^.

Os profissionais de saúde que mais orientaram as pessoas com DM sobre a importância de se vacinar foram os ACS e os enfermeiros. Ressalta-se que são esses profissionais que têm um contato mais frequente com pessoas com DM, e devem aproveitar o momento oportuno para orientá-los acerca dos benefícios de manter o esquema vacinal atualizado.

Destaca-se a importância de uma comunicação eficaz, que não apenas transmita informações sobre a importância da vacinação como medida preventiva, mas também promova a segurança das pessoas em relação às vacinas. Além disso, é fundamental fortalecer a credibilidade do PNI junto à população^(26)^, para evidenciar os benefícios da vacinação, bem como esclarecer dúvidas que impactam a decisão de se vacinar.

Entre os motivos para a incompletude do esquema vacinal, citam-se a falta de compreensão sobre a importância da vacinação e a ausência de informações fornecidas pelos profissionais. Sabe-se que tais agentes podem ser facilitadores para adesão à vacinação, incentivando o processo e tranquilizando as pessoas quanto à segurança e efeitos adversos^(9)^. Pesquisa^(26)^ apontou que o medo de eventos adversos compromete o acesso a vacinação, além de outros fatores socioeconômicos e geográficos. O entendimento desses fatores é essencial para o planejamento de estratégias de vacinação adaptadas às disparidades existentes e à necessidade de políticas mais equitativas nesse contexto.

Dessa forma, é fundamental intensificar campanhas, dispor de elucidações e oportunizar a prática de vacinação entre as pessoas usuárias, especialmente a população adulta jovem, que é mais resistente a procurar os serviços de saúde. Nessa perspectiva, destaca-se o papel do enfermeiro como educador em saúde, estabelecendo canais de comunicação para capilarizar informações sobre a vacinação e potencializar a respectiva adesão^(27)^.

Para todas as vacinas, a maioria da população com DM foi vacinada em estabelecimentos de saúde do SUS, sendo as USF os locais mais representativos. Isso consolida o PNI como um programa de acesso universal e fundamental para a saúde pública, garantindo que imunobiológicos essenciais estejam disponíveis para toda a população, independentemente de sua condição socioeconômica ou geográfica. Além disso, relaciona-se o fato da ampliação da cobertura populacional pela APS.

Entre os obstáculos para a vacinação, os mais relatados foram as dificuldades de deslocamento devido a limitações físicas ou falta de transporte, bem como a escassez de insumos ou vacinas no momento da imunização. Nota-se que essa população é predominantemente composta por pessoas idosas, muitas das quais enfrentam baixa renda e algum grau de limitação física devido ao envelhecimento, o que dificulta o acesso aos serviços de saúde.

Diante disso, estabelecimentos de saúde, especialmente na APS, devem implementar estratégias para alcançar pessoas com dificuldades de acesso, bem como articular serviços de atenção domiciliar para garantir a oferta de vacinação. Outro elemento identificado que dificultou o acesso a vacinação foi a falta de insumos/vacinas, sendo imprescindível planejar o quantitativo de doses necessárias. Aspectos relacionados a organização e planeamento de campanhas de vacinação podem influenciar a adesão de pessoas usuárias^(9)^.

As limitações do estudo referem-se à realidade local de realização da pesquisa, o que dificulta a generalização dos resultados para outras regiões. Dessa forma, são recomendadas novas pesquisas a fim de conhecer outros cenários, bem como os entraves relacionados à prática de vacinação na população com DM.

Acerca dos avanços ao conhecimento científico, trata-se do primeiro estudo nacional que analisou a situação vacinal de pessoas com DM para as vacinas recomendadas pelo PNI^(5-6)^, incluindo contra a covid-19, bem como trouxe suas percepções acerca da vacinação e as dificuldades enfrentadas na APS.

Assim, o presente estudo permitiu tecer reflexões acerca da importância da vacinação entre essas pessoas, considerando o estímulo aos profissionais de saúde, especialmente enfermeiros, para orientarem sobre a relevância e criarem momentos oportunos de vacinação, a exemplo de visitas para a realização de procedimentos como glicemia capilar. Ainda, espera-se que o estudo direcione intervenções para as causas da baixa completude vacinal, como a falta de orientação profissional, contemplando, inclusive, a adoção e o fortalecimento de práticas como o preenchimento do cartão de vacina da pessoa com DM e a utilização do eSUS-APS para registro de doses administradas.

## Conclusão

Verificou-se que pessoas com DM 1 e 2 apresentam baixa completude vacinal no contexto da APS, de acordo com o rol de vacinas recomendadas pelo PNI para tal grupo populacional. Salienta-se que são dados preocupantes, considerando o fato de serem pessoas em situação de maior vulnerabilidade a infecções imunopreveníveis e mortalidade, com agravamento da qualidade de vida caso desenvolvam uma doença passível de prevenção.

Recomenda-se reunir esforços para compreender os desafios de acesso à vacinação e melhorar a situação vacinal nas pessoas com DM por meio da educação permanente dos profissionais de saúde e aumento da conscientização pública. Destaca-se a atuação da equipe de enfermagem durante todo o processo de operacionalização das ações de vacinação no país, que pode ser decisiva para melhorar a situação vacinal em pessoas com DM.
